# Cecropin anisaxin-2S has *in vitro* immunomodulatory, but not antiproliferative and antiviral properties

**DOI:** 10.3389/fimmu.2025.1567505

**Published:** 2025-05-05

**Authors:** Jovana Majstorović, Anush Arakelyan, Željka Trumbić, Jan Kamiš, Mikolaj Adamek, Tomislav Rončević, Vedrana Čikeš Čulić, Jiří Kyslík, Martin Palus, Ivan Fiala, Ivona Mladineo

**Affiliations:** ^1^ Laboratory of Fish Protistology, Institute of Parasitology, Biology Centre Czech Academy of Sciences, Ceske Budejovice, Czechia; ^2^ Faculty of Science, University of South Bohemia, Ceske Budejovice, Czechia; ^3^ Laboratory of Functional Helminthology, Institute of Parasitology, Biology Centre Czech Academy of Sciences, Ceske Budejovice, Czechia; ^4^ University Department of Marine Studies, University of Split, Split, Croatia; ^5^ Laboratory of Arbovirology, Institute of Parasitology, Biology Centre Czech Academy of Sciences, Ceske Budejovice, Czechia; ^6^ Fish Disease Research Unit, Institute for Parasitology, University of Veterinary Medicine Hannover, Hannover, Germany; ^7^ Department of Biology, Faculty of Science, University of Split, Split, Croatia; ^8^ Department of Medical Chemistry and Biochemistry, University of Split School of Medicine, Split, Croatia; ^9^ Institute for Marine and Antarctic Studies, University of Tasmania, Hobart, TAS, Australia

**Keywords:** anisaxin, antimicrobial peptide, red blood cells, white blood cells, immunomodulation

## Abstract

Helminthic host defense peptides (HDP) are pleiotropic, multifunctional effector molecules of helminth immunity, efficient against Gram-negative and Gram-positive bacteria. Among them, anisaxin-2S (A-2S), membranolytic cecropin-like HDPs produced by the zoonotic nematodes of the genus *Anisakis*, shows remarkable efficacy even against multidrug-resistant Gram-negative bacteria, yet its immunomodulatory, antiproliferative and antiviral properties have not been elucidated. Therefore, we tested A-2S immunomodulation in the common carp (*Cyprinus carpio*) blood cells exposed to two pathogens, the zoonotic bacterium *Aeromonas hydrophila* and the fish parasite *Sphaerospora molnari*, and in carp *in vivo* challenged with the parasite. Furthermore, the A-2S antiproliferative activity was tested *in vitro* in human bladder and lung cancer cell line, while the antiviral protection was tested in common carp brain cell culture exposed to carp rhabdovirus, alloherpesvirus and paramyxovirus, and in a human immortalized myelogenous leukemia cell line infected with tick-borne encephalitis virus. A-2S exerts an immunostimulatory effect on fish blood cells through upregulation of cytokine expression, with the proinflammatory or anti-inflammatory repertoire conditioned by the presence or absence of co-stimulatory antigen. Surprisingly, in the majority of assays conducted, red blood cells demonstrate equal or even stronger regulation of innate immunity genes compared to white blood cells, along with a more extensive repertoire of differentially expressed markers. In contrast, A-2S has only a limited anticancer activity in human bladder cancer and lung adenocarcinoma cells and limited antiviral activity against the three fish viruses and a human tick-borne encephalitis virus. This study provides the first evidence of red blood cell and platelet immunomodulation by an antimicrobial peptide and highlights the induction of a cytokine repertoire. However, future research should address the study’s limitations, including the need for longer *in vitro* assays (e.g., 3–4 days), testing different white blood cell lineages, to better understand antigen-processing interactions, and evaluating the anticipated adaptive immune response. Powerful antimicrobial activity of A-2S, coupled with immunostimulatory properties, warrant further pursuing of preclinical trials with this anisaxin.

## 1 Introduction

Antimicrobial activity, primarily associated with immunomodulatory properties, is a common feature of a heterogeneous group of peptides known as host defense peptides (HDP), whereas the ones with direct antimicrobial properties are also referred to as antimicrobial peptides (AMP). Structurally differentiated as α-helix, β-sheet, cysteine bridge-stabilized CSαβ and non-αβ peptides with extended conformation (1), HDPs are multifunctional and pleiotropic effector molecules of innate immunity, capable of modulating immune cells activity in inflammatory processes and stimulating cell proliferation in angiogenesis or wound healing (2). Their antimicrobial activity has been found against Gram-negative and Gram-positive bacteria, fungi, viruses and parasites, especially Protozoa (3). HDPs are expressed by epithelial and immune cells of invertebrates and vertebrates, although they are also being produced by other phyla throughout the tree of life, including bacteria, fungi, algae, and plants.

Currently, only six anti-infective peptides are in clinical use in medicine (e.g., daptomycin and gramicidin D), despite hundreds of therapeutic peptides and proteins encompassed in The Therapeutic Protein Database and approximately 60 peptide-based drugs listed in Drug Bank 5.0 (4). There are multiple reasons for this: i) development of transient resistance in bacteria; ii) necessity for AMP encapsulation in carriers, or cyclization, amidation and incorporation of non-proteinogenic amino acids to stabilize AMP structure in host fluids; iii) manufacturing costs related to chemical synthesis, and iv) toxicity as a side effect toward host cells. Interestingly, HDPs excreted and secreted by parasitic helminths have recently gained more interest due to their potentially lower toxicity to the host cell, which is usually the main obstacle to the therapeutic use of HDPs from the free-living organisms (4). In helminths, HDPs are thought to facilitate the communication and interaction among the helminth, the host and the surrounding microbiota. However, it is difficult to discern whether these interactions exert a direct antimicrobial effect, or rather modulate the innate immunity of the host, which consequently exerts increased antimicrobial activity against the pathogens. Interestingly, parasitic flatworms have been observed to benefit from HDPs with mainly immunomodulatory properties (5–7), while nematodes possess HDPs with pronounced direct antimicrobial activity. This potential specialization of HDPs could be related to evolutionary differences between helminths phyla, but it remains to be confirmed yet in a larger number of species. However, in most cases, both flatworms and nematodes colonize the same intestinal niche within the host, suggesting that factors other than the site of parasitation play a role in the specialization of HDPs (4).

Cecropin-like peptides called anisaxins have recently been identified and characterized in the zoonotic marine nematodes *Anisakis simplex* and *Anisakis pegreffii* that use fish as paratenic, marine mammals as final, and humans as accidental hosts (8). The nematodes express them as pre-propeptides, which in mature form are cationic and moderately amphipathic α-helical peptides. All five characterized isoforms show strong antibacterial activity against Gram-negative bacteria at sub micromolar or low micromolar concentrations, including multidrug-resistant (MDR) isolates. As typical cecropins, anisaxins are membranolytic, extracting the lipids from the bacterial membrane, ultimately leading to cell leakage and bacterial death. The antimicrobial, physico-chemical and pharmacokinetic properties of anisaxins and to some extent their mode of action have been characterized in more detail in comparison to other helminthic HDPs, providing a robust model for studying other related bioactive properties. In addition, A-2S has shown remarkably low cyto- and genotoxicity in human cells, which makes it an attractive candidate for further pre-clinical trials in humans. However, other biological properties, such as immunomodulatory, antiproliferative and antiviral effects have to be evaluated to ensure safe application in trials. The aim of this study was therefore to investigate whether anisaxin-2S (A-2S), the most potent of the anisaxins against Gram-negative bacteria, also possesses: i) immunomodulatory activity in fish (paratenic host) immune cells either *in vitro* or *in vivo* during pathogen challenge, ii) *in vitro* antiproliferative properties in human (accidental host) urinary bladder and lung cancer cell lines, and iii) *in vivo* antiviral activity against fish and human viruses.

## 2 Results

### 2.1 Gene expression of RBC and WBC upon *in vitro Aeromonas hydrophila* and A-2S stimulation

**Figure 1:**
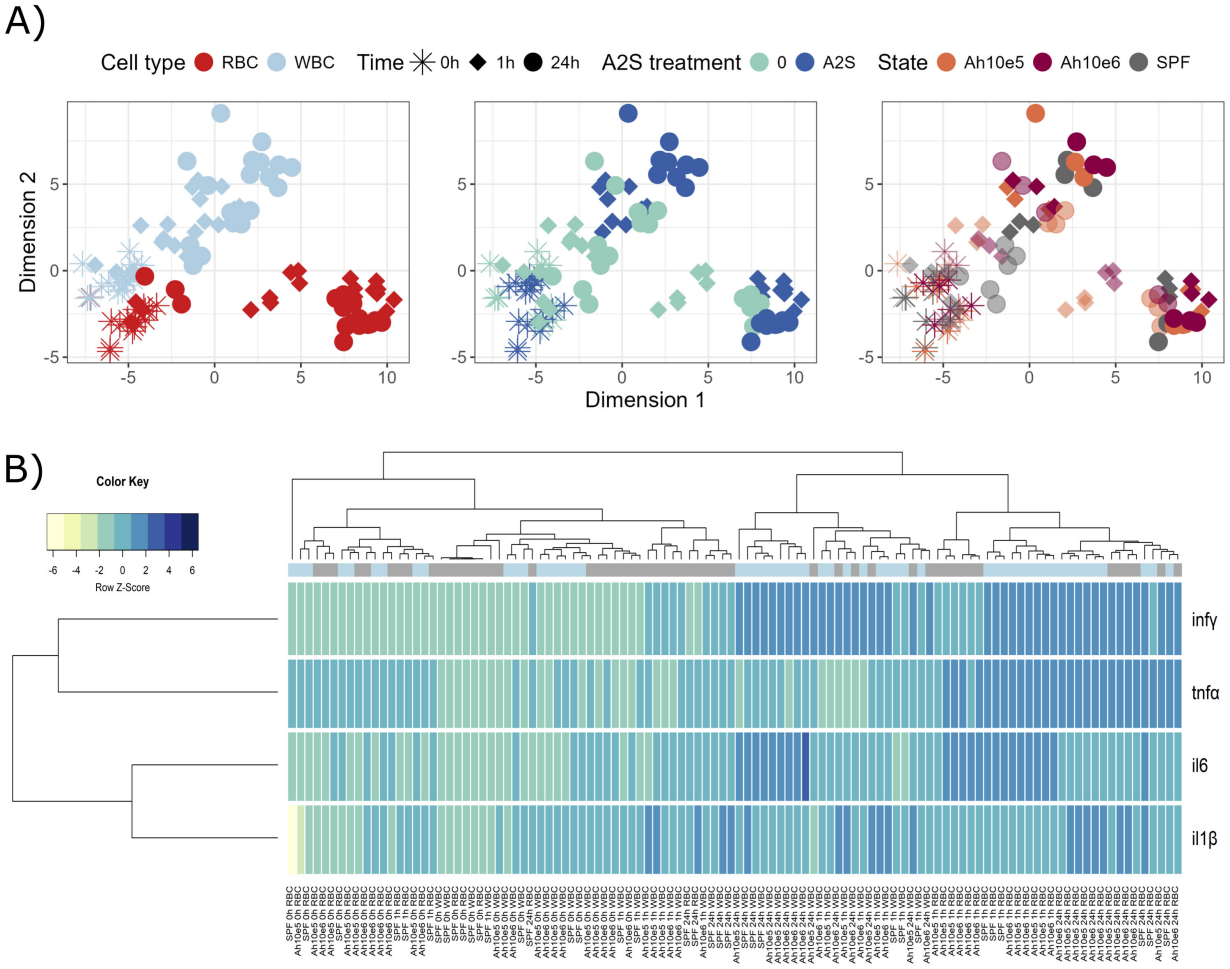
Graphical representations of expression profiles of cytokines after *in vitro* stimulation (0, 1 and 24 h) of red blood cells (RBC) and white blood cells (WBC) of *Cyprinus carpio* with *Aeromonas hydrophila* (1 x 10^5^ and 1 x 10^6^) and/or anisaxin A-2S antimicrobial peptide (10 µM). Data were normalized to housekeeping gene and log2 transformed. **(A)** Differences/similarities between samples after treatment were shown using multidimensional scaling. Different experimental aspects (i.e., difference between RBC vs WBC over time, A-2S treated vs untreated cells over time, and two *A. hydrophila* concentrations vs untreated cells over time) are shown using color in separate panels. A-2S treatment in the third panel is indicated by full coloring and untreated samples by transparent colors. **(B)** Expression profiles of target genes were clustered and displayed using a heatmap. A-2S treatment is indicated in light blue boxes under sample dendrogram and in gray are unstimulated cells. Color key legend denotes a Row Z-Score of standard deviation of a target gene from -6 to 6 from the mean expression level across all samples. SPF are cells from specific pathogen-free carp which served as negative control. Ah10e5: *Aeromonas hydrophila* 1 x 10^5^ treated cells, Ah10e6: *Aeromonas hydrophila* 1 x 10^6^ treated cells, SPF: specific pathogen-free cells (untreated), inf*γ* : interferon *γ*, tnf*α*: tumor necrosis factor *α*, il6: interleukin-6, il-1*β*: interleukin-1*β*.

Isolated fish blood cells were *in vitro* stimulated by two different concentrations of the bacterial pathogen *A. hydrophila*, with or without addition of A-2S, and the expression of immune genes in response to the stimulation was measured. During *A. hydrophila* and A-2S stimulation, red blood cells (RBC) and white blood cells (WBC) display different temporal dynamics of gene expression changes. A-2S induces changes in both RBCs and WBCs, causing treated group of samples to shift away from their untreated counterparts, somewhat more in WBC than in RBC (**Figure 1A**). Samples primarily cluster according to their cell type and hour of stimulation and less according to bacterial treatment (**Figure 1B**). Generally, *infγ*  (interferon *γ*) is upregulated in RBC and WBC under A-2S treatment with time, *tnfα* (tumor necrosis factor *α*) is more characteristic for RBC response and *il6* (interleukin-6) in WBC (**Figure 1B**). Specifically, A-2S induces upregulation of *il6*, *tnf*α** and *inf*γ** in control RBC (not exposed to *A. hydrophila* antigen) either 1 or 24 h after stimulation but does not significantly stimulate cytokine expression in cells already exposed to a concentration of 1 x 10^5^ or 1 x 10^6^ *A. hydrophila*. Expression of *il-1β* (interleukin-1*β*) is not observed at any time point and with any treatment (**Supplementary Figure S1A**, **Supplementary Table S1**).

Similarly, in control WBC, A-2S upregulates the expression of *il6* at 24 h and of *infγ* at 1 and 24 h post-stimulation. It also affects the upregulation of *il-1β* at 24 h in cells incubated with 1 x 10^6^ *A. hydrophila*. Expression of *tnfα* is not observed at any time point and with any treatment (**Supplementary Figure S1B**, **Supplementary Table S1**).

When comparing RBC cytokine expression among time points (0, 1 and 24 h) within a given treatment, only *il6* was already significantly upregulated at 1 h (with few exceptions). *tnfα* was mainly upregulated at 24 h, and *infγ*  only at 24 h in A-2S-stimulated cells (**Supplementary Figure S1C**, **Supplementary Table S1**).

In WBC, statistically significant expression at either 1 or 24 h was observed only for *infγ* within most treatments, while *il-1β* showed increased expression only at 24 h after treatment with 1 x 10^6^ *A. hydrophila* (**Supplementary Figure S1D**, **Supplementary Table S1**).

### 2.2 Gene expression of RBC, WBC and TC upon *in vitro Sphaerospora molnari* and A-2S stimulation

**Figure 2:**
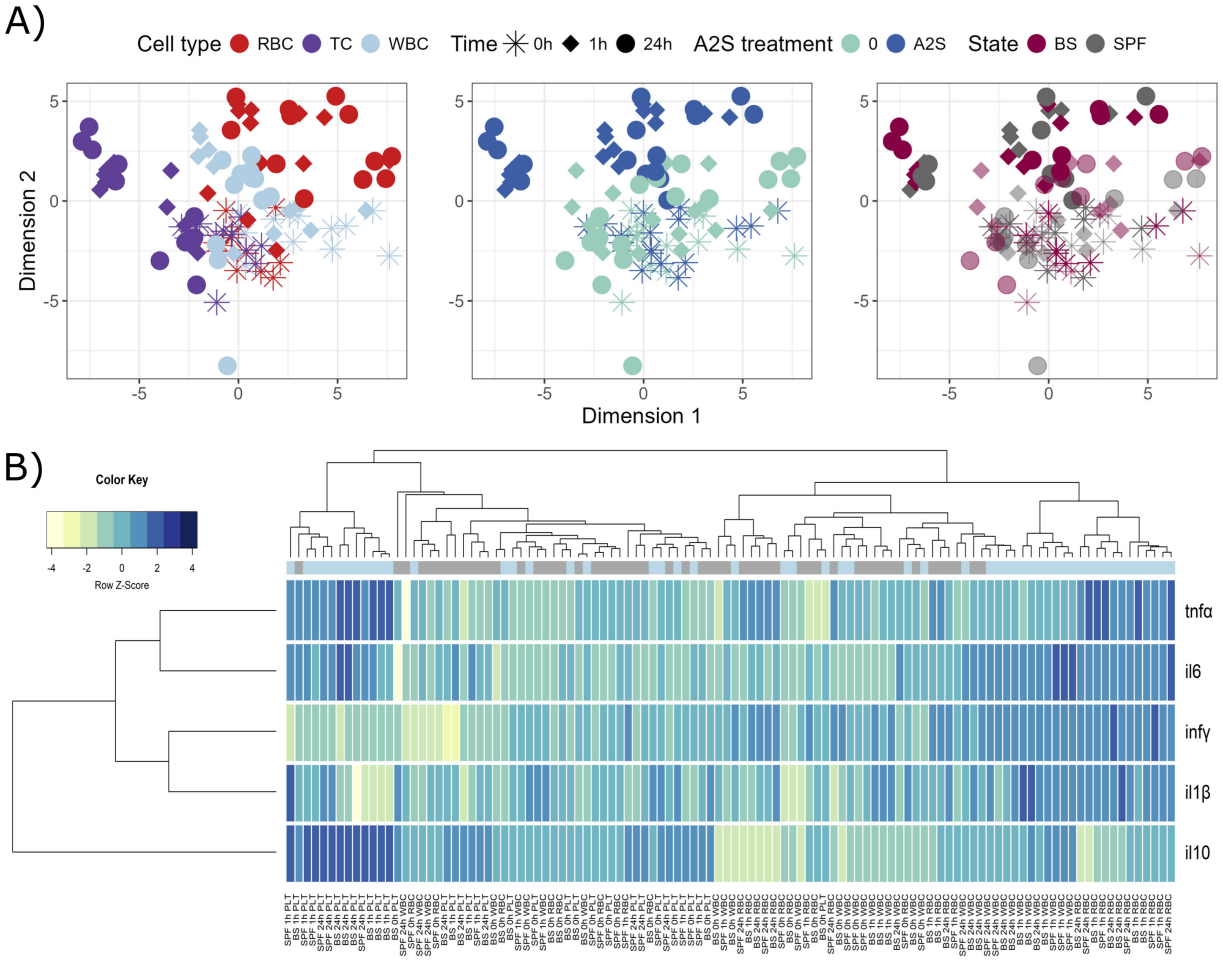
Graphical representations of expression profiles of cytokines after *in vitro* stimulation (0, 1 and 24 h) of red blood cells (RBC), white blood cells (WBC) and platelets (TC) of *Cyprinus carpio* with *Sphaerospora molnari* blood stages (BS) and/or anisaxin A-2S antimicrobial peptide (10 µM). Data were normalized to housekeeping gene and log2 transformed. **(A)** Differences/similarities between samples after treatment were shown using multidimensional scaling. Different experimental aspects (i.e., differences among RBC, TC and WBC over time, A-2S treated vs untreated cells over time, and cells from *Sphaerospora molnari*-infected vs SPF carp over time) are shown using color in separate panels. A-2S treatment in the third panel is indicated by full coloring and untreated samples by transparent colors. **(B)** Expression profiles of target genes were clustered and displayed using a heatmap. A-2S treatment is indicated in light blue boxes under sample dendrogram and in gray are unstimulated cells. Color key legend denotes a Row Z-Score of standard deviation of a target gene from -4 to 4 from the mean expression level across all samples. SPF are cells from specific pathogen-free carp which served as negative control. inf*γ* : interferon *γ*, tnf*α*: tumor necrosis factor *α*, il6: interleukin-6, il-1*β*: interleukin-1*β*.

Isolated fish blood cells were *in vitro* stimulated by a parasitic pathogen *S. molnari*, with or without addition of A-2S, and the expression of immune genes in response to the stimulation was measured. Similar to the previous assay, different blood components, i.e., RBC, WBC and thrombocytes (TC), respond differently to parasite and A-2S stimulation, causing samples to diverge in different directions, especially platelets (**Figure 2A**). There are two clusters in heatmap showing general separation of A-2S-treated platelets at 1 h and 24 h on one hand, and A-2S-treated WBC and RBC at 1 h and 24 h on the other (**Figure 2B**). Although the separation is not perfect, it seems that platelets response under A-2S is generally characterized by increased *il10* (interleukin-10) expression and reduced expression of *infγ*, which is the opposite in RBC and WBC. Two cytokines, *tnfα* and *il6* seem to be induced in all conditions, while *il-1β* only in RBC, and WBC. More specifically, A-2S induces an upregulation of *il6*, *tnfα* and *infγ* in RBC of healthy SPF fish either at 1 h, but mainly 24 h post-stimulation, and it significantly stimulates the expression of *il-1β* only in cells already exposed to *S. molnari* (**Supplementary Figure S2A**, **Supplementary Table S2**).

In WBC, *il6* is also upregulated at 1 and 24 h post-stimulation and *infγ* is upregulated in untreated cells (SPF) and *S. molnari*-stimulated cells. A-2S also influences the upregulation of *il-1β* at 24 h in cells exposed to *S. molnari* (**Supplementary Figure S2B**, **Supplementary Table S2**).

When cytokines expression of RBC was compared among time points (0, 1 and 24 h) within a given treatment, all genes except *tnfα* were significantly upregulated already after 1 h in all A-2S-stimulated cells. The exception was *il6*, which was not significantly upregulated in SPF cells stimulated by A-2S. *tnfα* was mainly upregulated after 24 h (**Supplementary Figure S2C**, **Supplementary Table S2**).

In WBC, statistically significant expression was observed for all cytokines under most treatments at either 1 or 24 h, while *tnfα* only showed increased expression in A-2S-stimulated cells at 1 h post-treatment (**Supplementary Figure S2D**, **Supplementary Table S2**). Platelets stimulated by A-2S upregulated *il6* (only when cells were not exposed to *S. molnari*) and *tnfα* (even in case when exposed to *S. molnari*) (**Supplementary Figure S3A**, **Supplementary Table S2**). The difference between time points was most pronounced for *il6*, which was expressed in cells under all treatments (**Supplementary Figure S3B**, **Supplementary Table S2**). Interestingly, the expression of the anti-inflammatory cytokine *il10* was significantly upregulated only in platelets stimulated by A-2S.

### 2.3 Gene expression of RBC and WBC of *Sphaerospora molnari in vivo*-challenged fish and *in vitro* stimulated with A-2S

**Figure 3:**
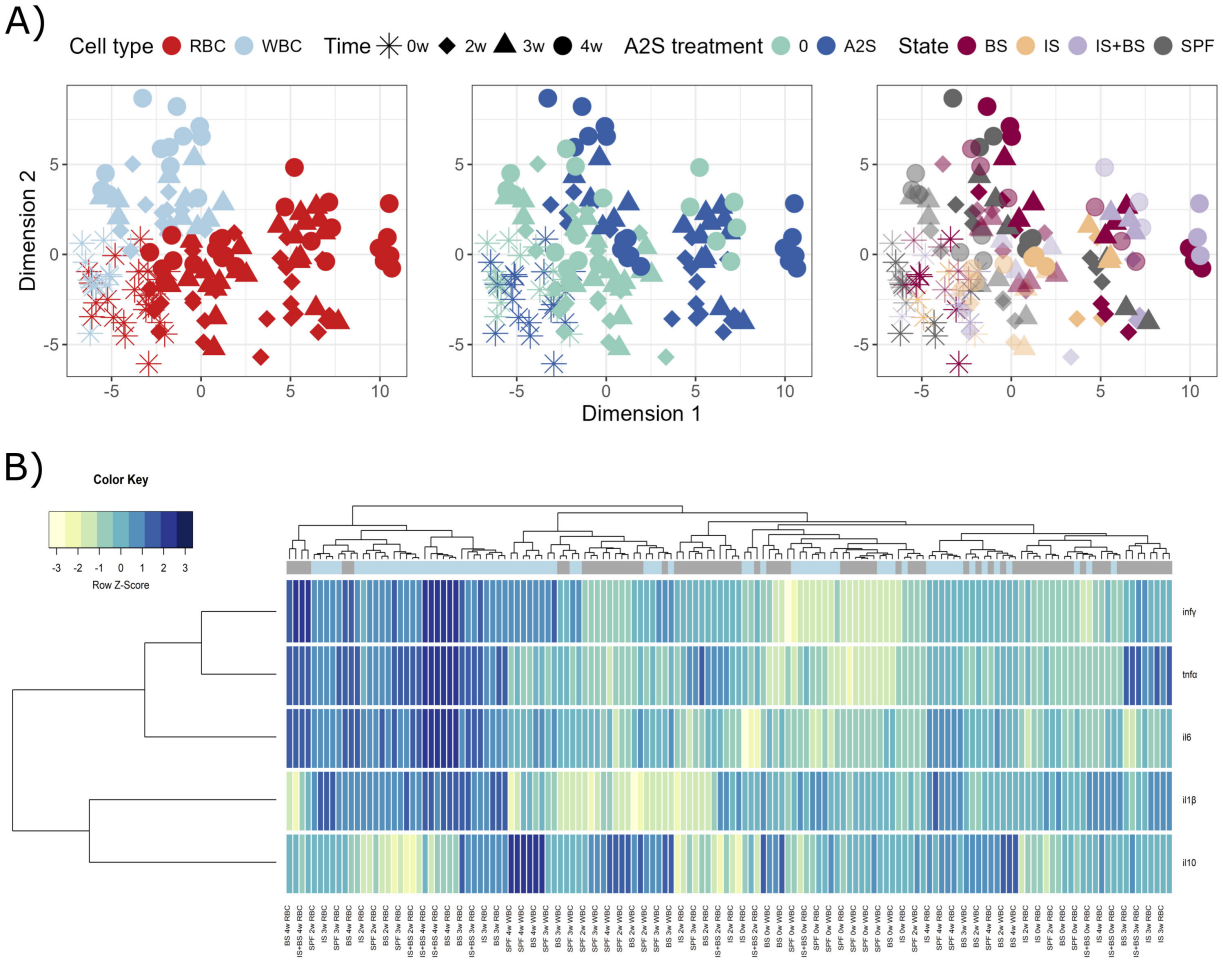
Graphical representations of expression profiles of cytokines in red blood cells (RBC) and white blood cells (WBC) of *Cyprinus carpio* priorly *in vivo* challenged by *Sphaerospora molnari* blood stages (BS), immunosuppressed (IS), or both (IS+BS). SPF are specific pathogen-free carp used as negative control. After collection of RBC and WBC from carp at specific time-points (0, 2, 3 and 4 weeks), the cells were stimulated or non-stimulated with anisaxin A-2S antimicrobial peptide (10 µM). Data were normalized to housekeeping gene and log2 transformed. **(A)** Differences/similarities between samples after treatment were shown using multidimensional scaling. Different experimental aspects (i.e., differences between RBC vs WBC over time, A-2S treated vs untreated cells over time, and among cells from *Sphaerospora molnari*-infected, immunosuppressed, immunosuppressed and *S. molnari*-infected and SPF carp over time) are shown using color in separate panels. A-2S treatment in the third panel is indicated by full coloring and untreated samples by transparent colors. **(B)** Expression profiles of target genes were clustered and disapplied using a heatmap. A-2S treatment is indicated in light blue boxes under sample dendrogram and in gray are unstimulated cells. Color key legend denotes a Row Z-Score of standard deviation of a target gene from -3 to 3 from the mean expression level across all samples. inf*γ* : interferon *γ*, tnf*α*: tumor necrosis factor *α*, il6: interleukin-6, il-1*β*: interleukin-1*β*.

Fish were firstly experimentally infected with the parasitic pathogen *S. molnari*, then at different time points, their blood cells were isolated and stimulated *in vitro* with different concentrations of the A-2S, and the expression of immune genes in response to the stimulation was measured. After prolonged exposure to *S. molnari* blood stages, immunosuppression or both, followed by A-2S *in vitro* stimulation, RBC and WBC demonstrate different responses (**Figure 3A**). Response to A-2S stimulation seems to be stronger in RBC, however it was not specific for any particular treatment. After 2, 3 or 4 weeks of exposure RBC generally upregulated all tested genes, except *il10*, which is more pronounced in WBC, together with lesser regulation of *il-1β* (**Figure 3B**). Specifically, The RBC of healthy fish stimulated with A-2S alone showed no significant cytokine upregulation during the 4-week experiment. In contrast, in fish immunosuppressed alone or in combination with *S. molnari* infection, *il6* and *tnfα* were upregulated throughout the experiment. Upon A-2S stimulation, *infγ* was significantly upregulated only at week 2 of infection in immunosuppressed fish and *il-1β* at the onset of parasitemia (week 4) in SPF and *S. molnari*-infected fish (**Supplementary Figure S4A**, **Supplementary Table S3**).

In the WBC of healthy fish, *tnfα* (week 3) and *infγ* (weeks 2 and 3) were significantly upregulated upon A-2S stimulation. The same cytokines were upregulated in fish in the stage of parasitemia (**Supplementary Figure S4B**, **Supplementary Table S3**). Interestingly, the expression of the anti-inflammatory cytokine *il10* was significantly upregulated only in the WBC in the parasitemia stage (**Supplementary Figure S5**).

All cytokines in the RBC were differentially upregulated over time (0, week 2-4), but the most intense changes were measured in *infγ* (**Supplementary Figure S4C**, **Supplementary Table S3**).

Similar was observed in the WBC, except that there was no differential expression of *il6*. Interestingly, the expression of *il-1β* in WBC was downregulated by the shift in the parasitemia in infected fish, either when stimulated with A-2S or not (**Supplementary Figure S4D**, **Supplementary Table S3**).

### 2.4 Cellular changes in *A. hydrophila* and *S. molnari* blood stages upon A-2S treatment

**Figure 4:**
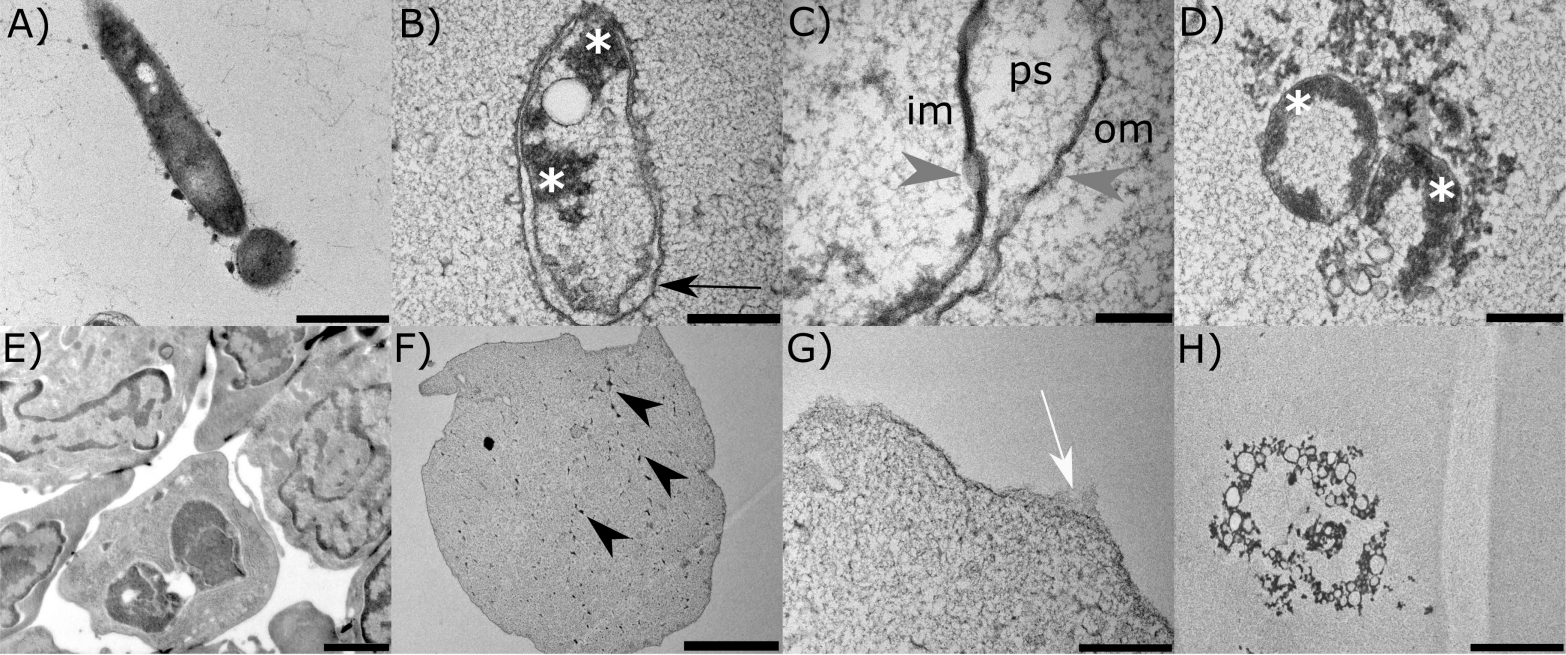
Representative transmission electron micrographs of the two pathogens of the common carp (*Cyprinus carpio*); bacterium *Aeromonas hydrophila* and the parasite *Sphaerospora molnari* treated *in vitro* with anisaxin A-2S (10 µM). Untreated bacterium and parasite are shown in **(A, E)**. In the former **(B)**, A-2S induces vacuolization of the cytoplasm and plasmolysis, chromatin condensation and accumulation at the periphery (white *). The outer cell wall membrane (om) detaches from the inner cell wall membrane (im), forming periplasmatic spaces (ps) **(C)**. The cell wall membranes have a “smudged” appearance (gray arrowheads). Fragmentation of bacteria is also observed **(D)**. Parasite cells **(F)** are plasmolytic, with fragmented nuclei (black arrowheads), spongy cytoplasm, and disrupted cell membrane **(G)**. Leftovers of necrotic cells are also observed **(H)**.

Pathogens *A. hydrophila* and *S. molnari* were *in vitro* treated by A-2S to observe the changes in their ultrastructure. Compared to control *A. hydrophila* (**Figure 4A**), cells treated with 10 µm A-2S (approximately 10x MIC) show vacuolization of the cytoplasm and plasmolysis, chromatin condensation and its accumulation at the cell periphery (**Figure 4B**). The outer cell wall membrane is irregular, with blurred projection and detachment from the inner cell wall membrane, resulting in wide periplasmic spaces. There are no distinctive breaks in the continuity of either the outer or inner cell wall. Instead, the cell wall membranes are thinned and have a “smudged” appearance (**Figure 4C**). Fragmented bacteria are also observed (**Figure 4D**).

The control blood stages of *S. molnari* are multinucleate forms consisting of secondary cells within a primary cell (**Figure 4E**). Cells treated with A-2S appear plasmolytic, with fragmented nuclei, spongy cytoplasm (**Figure 4F**) and ruptured cell membrane (**Figure 4G**). Remnants of cells in necrosis are also observed (**Figure 4H**).

### 2.5 ROS production in RBC and WBC upon A-2S stimulation

WBC from fish *in vivo* challenged with *S. molnari* blood stages, and RBC from *in vivo* challenged and immunosuppressed fish, both collected at week 4 and treated with A-2S were assayed for the production of reactive oxygen species (ROS). In all cases, a statistically significant increase in numbers and increase in production of ROS (**Supplementary Figures S6, S7**).

The RBC count monitored over 4-weeks period (without subsequent A-2S *in vitro* treatment) of the challenged fish begun to decrease significantly in the 3rd week of infection, by approximately 10% (100 x 10^6^ RBC/mL) compared to the total cell count. In fish that were challenged and immunosuppressed, the decrease from the 3rd week onwards led to an 84% decrease in RBC count at the end of the experiment. Immunosuppressed fish showed a slight increase in RBC compared to the control and toward the end of the experiment, due to an increase in erythroblast lineage accompanied by the decrease in mature erythrocytes.

### 2.6 Antiproliferation in human cancer cell lines upon A-2S stimulation

**Figure 5:**
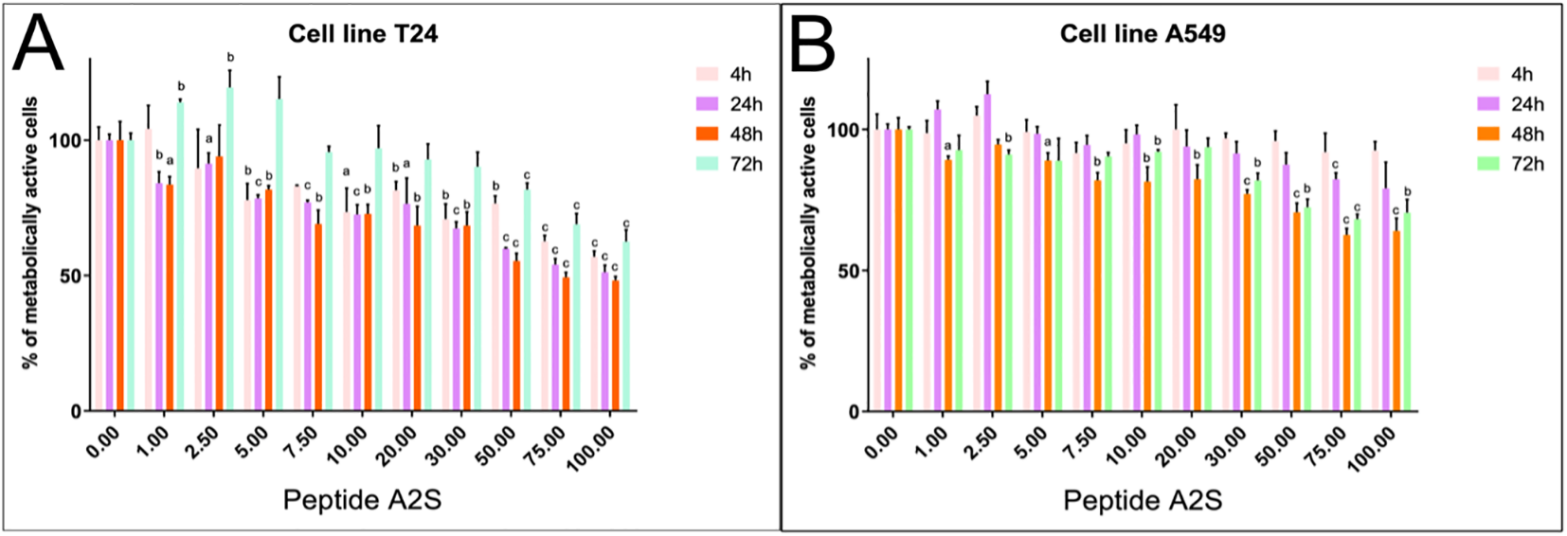
Percentage of metabolically active human bladder cancer cell line T24 **(A)** and human lung cancer cell line A549 **(B)** treated by a range of A-2S concentrations (1, 2.5, 5, 7, 10, 20, 30, 50 and 100 µM) 4, 24, 48 and 72 h post-treatment. Each sample was measured in triplicate. Statistically significant difference is expressed at P<0.05 **(a)**, P<0.01 **(b)** and P<0.001 **(c)**.

To assess whether A-2S has anti-cancerogenic properties, the cecropin has been applied in two human cancer cell lines. A-2S led to a better arrest of cell proliferation in the human bladder cancer cell line T24 (**Figure 5A**) than in the lung cancer cell line A549 (**Figure 5B**). For the latter, the IC50 for each time points were incalculable. The IC50 for the T24 cell line was incalculable at 4 h, 68.45 µM at 24 h, 56.47 µM at 48 h, and incalculable at 72 h. Both cell lines showed the best antiproliferative activity after 48 h of treatment, but then decreased after 72 h.

### 2.7 Virucidal effect of A-2S in fish and human viruses

Virucidal properties of A-2S have been tested in fish and human viruses. No statistically significant effect of A-2S on the infectivity of fish rhabdovirus (SVCV), alloherpesvirus (KHV) and paramyxovirus (CCPV) was observed. A-2S did not show even minimal virucidal activity; the effect of A-2S at concentrations of 100 µM, 50 µM and 10 µM was within one standard deviation of TCID50 for untreated viruses.

**Figure 6:**
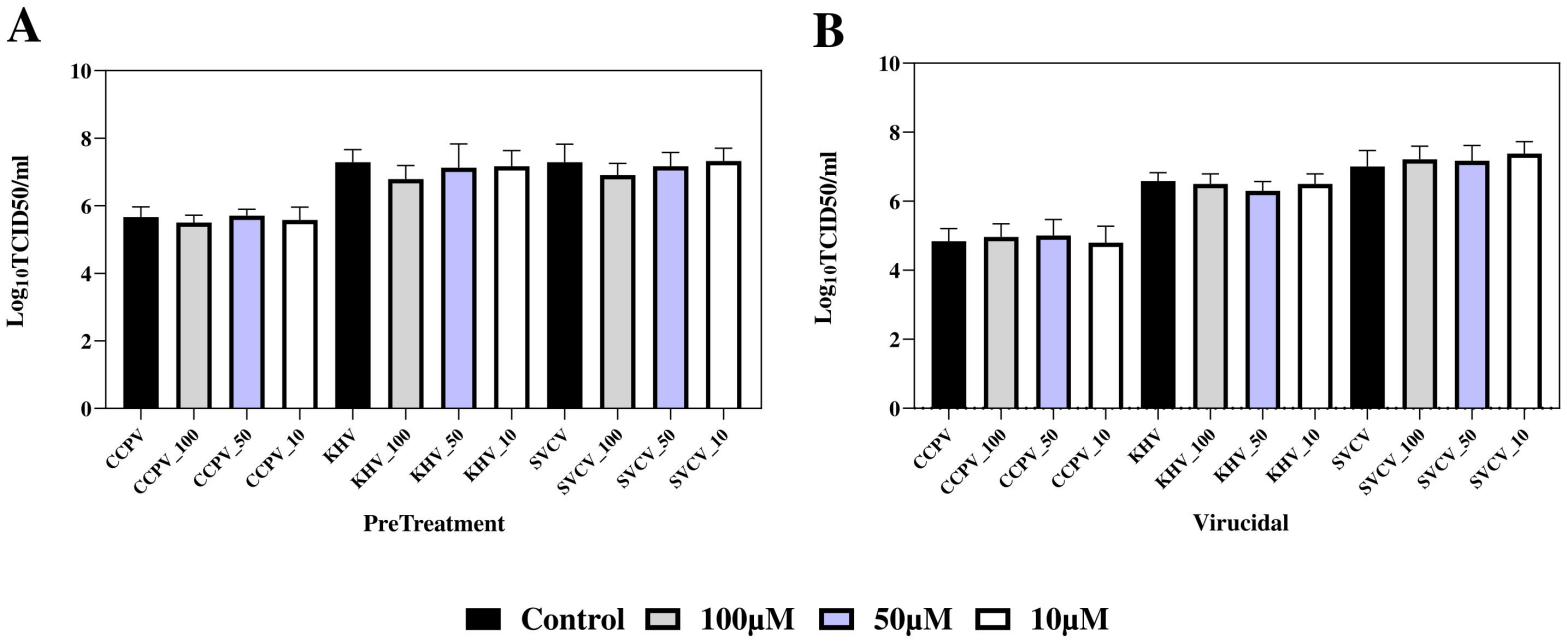
Preventive (PreTreatment, **A**) and virucidal (Virucidial, **B**) activity of three A-2S concentrations (10, 50 and 100 µM) in common carp brain (CCB) cell line infected by common carp paramyxovirus (CCPV), koi herpesvirus (KHVC) and spring viremia of carp virus (CSVCV) expressed as a log transformed mean of six replicates of 50% tissue culture infective dose (Log_10_TCID50/mL). In control virucidal and control pre-treatment (black columns), A-2S was omitted.

When CCB monolayers were pre-incubated for 8 h with culture medium containing A-2S at a final concentration of 100 µM, a weak but statistically insignificant effect was observed, reducing the TCID50 of the viruses by 1.5-3-fold times. The lower concentrations of A-2S (50 µM and 10 µM) had no effect (**Figure 6**).

**Figure 7:**
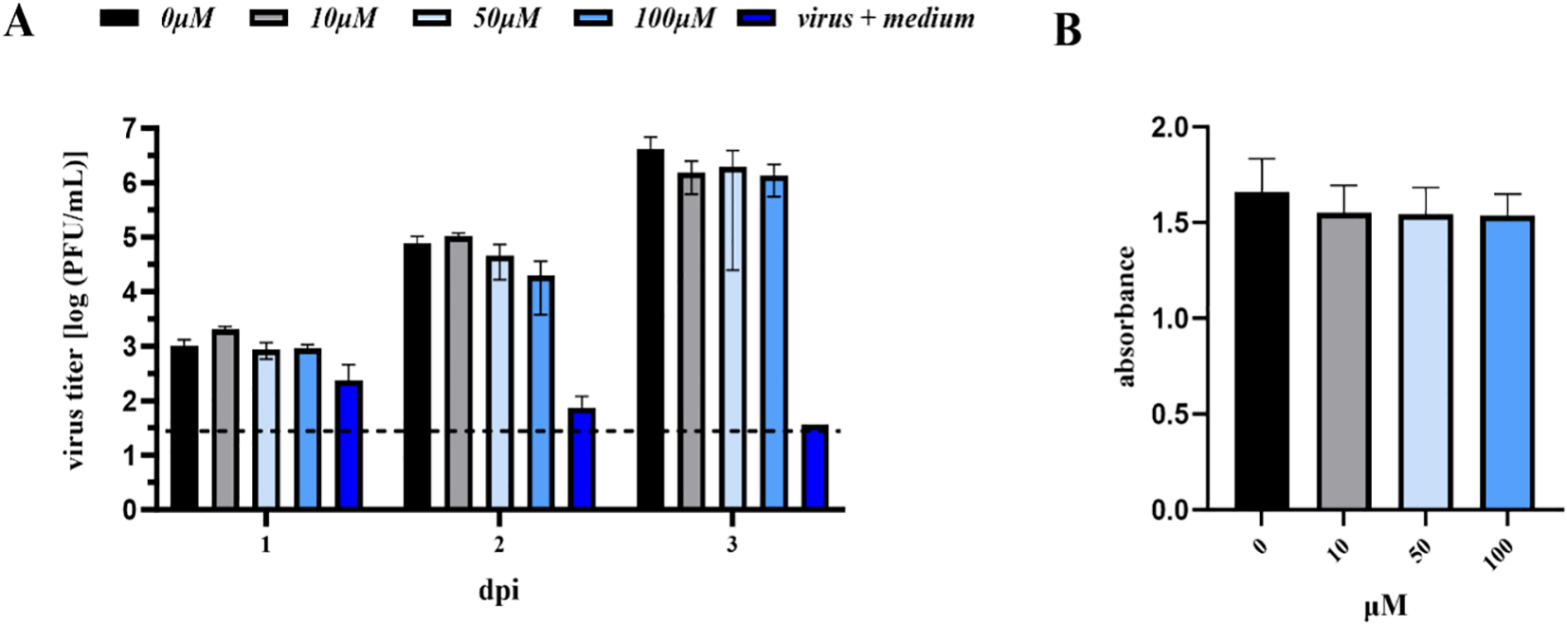
**(A)** Antiviral assay performed in the human lymphoblast cell culture K-562 (CCL-243) pre-treated with a range of A-2S concentrations (0, 10, 50 and 100 µM) and infected by TBEV Neudoerfl 0.01 MOI (Multiplicity of Infection). Virus titer (PFU/mL) was evaluated using a plaque assay 1-, 2- and 3-days post-infection (dpi). **(B)** Survival (expressed as absorbance) of human lymphoblast cell culture K-562 (CCL-243) treated by three concentrations of A-2S over 72 h evaluated in the MTT assay.

In the case of anti-TBEV assays, no statistically significant difference was observed between the untreated control cells and the cells treated with A-2S, i.e., A-2S concentrations up to 100 µM had no effect on the proliferation of K-562 cells, as shown by the MTT assay. Similarly, no effect was observed when K-562 cells treated with A-2S at all concentrations were infected with TBEV (**Figure 7**).

Interestingly, the K562 cell line stimulated by A-2S alone showed significantly higher expression of all three measured gene targets, *infα*, *tnfα* and *fcγr*, which showed increasing trend from 1 to 24 h, except for the former (**Supplementary Figure S8**).

## 3 Discussion

### 3.1 Immunomodulatory properties

Anisaxin-2S shows significant immunomodulatory properties in the blood cells of healthy fish. It initially enhances a pro-inflammatory response, which is balanced by a subsequent anti-inflammatory response. In contrast, in the presence of a co-antigen, i.e., fish infected with the bacterium *A. hydrophila* or the parasite *S. molnari*, the environment becomes anti-inflammatory at an early stage. The repertoire and extent of expression of innate immunity markers is highly dependent on the type of cell lineage exposed to A-2S (i.e., platelets, white, or red blood cells), the duration of infection (1 vs. 24 h, or 0 vs. 2, 3 and 4 weeks) and the general health status of the fish (i.e., parasite-infected, immunosuppressed, etc.). As a rule of thumb, the expression of selected markers of the innate immunity increases toward 24 h, or over four weeks, in the case of the *in vivo* challenge assay with *S. molnari*.

Unexpectedly, in most assays performed, RBC show equal or even a stronger regulation of measured innate immunity genes compared to WBC, as well as a broader repertoire of differentially expressed markers. The proinflammatory response begins with a striking early but transient *in vitro* upregulation (1 h) of *tnfα* in RBC co-stimulated by A-2S and both pathogens (*A. hydrophila* and *S. molnari*), suggesting a role for A-2S in activating the innate response of RBC via TLR9-NF-*κ*B axis, previously described by Hotz et al. (9) (discussed below). Tumor necrosis factor α is a pleiotropic cytokine affector of different cell types, and a major regulatory element of the inflammatory responses. Along another proinflammatory cytokine IL-1β, TNF-α is produced upon activation of the canonical NF-κB in various immune cells, as well as fish RBC. In the past, erythrocytes were considered as mere oxygen transporters and immunologically inert cells with minimal involvement in host defense, largely neglected as players in innate immunity (10). In mammalians, they have minimal capacity for transcription and protein synthesis due to loss of the nucleus during the maturation, although several immune functions have recently been demonstrated in RBC, e.g., the ability to modulate leukocyte activity, induce the release of cytokine-like factors, respond to pathogen-associated molecular patterns (PAMPs) and, in humans, express TLR9 on the surface (11). In contrast, fish RBC are nucleated and have a recognized role as cellular mediators of the immune response, ranging from the recognition of pathogens to their elimination by binding microbial immune complexes, and to the production of cytokines or other signaling molecules in response to pathogens (12). Furthermore, we demonstrated that A-2S-treated fish platelets, in addition to RBC, express *il6* and *tnfα* when co-stimulated with *S. molnari*. Mostly macrophages secrete IL-6 in response to specific PAMPs that bind to pattern recognition receptors (PRRs), including TLRs. IL-6 stimulates the acute phase protein synthesis, as well as the production of neutrophils in the bone marrow, the growth of B cells, antagonizing regulatory T cells. This is the first compelling evidence for blood cell elements other than WBC assuming the role of innate immunity when stimulated by a heterologous AMP.

*A. hydrophila* or A-2S applied to cells alone, induce, as expected, the expression of all examined proinflammatory markers (*il6*, *tnfα*, *infγ* 24 h post-stimulation) in RBC, supporting the proinflammatory role of A-2S. Among these, interferon *γ* is primarily secreted by CD^4+^ T helper 1 (Th1) cells, natural killer (NK) cells, and CD^8+^ cytotoxic T cells, activating the JAK-STAT signaling pathway and playing a role in both innate and adaptive immunity. In contrast, RBC stimulated simultaneously with the bacteria and the peptide showed no significant difference in gene expression (e.g., RBC stimulated by 10^5^ *A. hydrophila* vs. RBC stimulated by 10^5^ *A. hydrophila* and A-2S). Similarly, WBC are also unaffected when stimulated simultaneously with the bacteria and the peptide (with the exception of *il-1β*, which is expressed at higher *A. hydrophila* concentration for 24 h) and show no changes in *tnfα* expression. This anti-inflammatory milieu is also detected by the downregulation of *il6* after A-2S treatment when the peptide is administered with either bacterial concentration (at 24 h). This could be due to the interaction of A-2S with the LPS, which has been well described with other AMPs such as temporins and pardaxins. These AMPs interact with LPS, rearrange its structure and change the level of aggregation, resulting in individual LPS structures of smaller size (13, 14). The latter, in turn, bind less avidly to the LPS-binding protein, and prevent the expression of the proinflammatory repertoire induced by activation of Toll-like receptor 4 (TLR4), abrogating in particular *tnfα* expression (15), as observed here. Cathelicidins also block the binding of LPS to CD14, disaggregate LPS and remove LPS from the cell surface in monocyte- or macrophage-like cells, thereby inhibiting the production of proinflammatory cytokines (16). However, the exact signaling pathways affected by cathelicidins or other HDPs are poorly understood. It is assumed that in mammals, cathelicidin LL-37 binds to glyceraldehyde-3-phosphate dehydrogenase (GAPDH) to promote the anti-inflammatory response, via the p38 mitogen-activated protein kinase (MAPK) signaling pathway (17).

*In vitro* priming with the parasite and subsequent co-stimulation by A-2S resulted in a similar trend in the RBC as observed for the bacterium; no difference in the expression of most cytokines was observed between the cells treated with the parasite alone and those co-stimulated by the parasite and A-2S. The only exception was *il-1β*, which was upregulated for 24 h and was also the only cytokine upregulated in the RBC as early as 1 h after stimulation by A-2S alone. This indicates a more pronounced early pro-inflammatory response in the parasite *in vitro* assay. Interestingly, this opposing balance between the general anti-inflammatory environment and an early pro-inflammatory expression of *il-1β* was also observed for LL-37. It appears to be due to an interaction of phosphoinositide 3-kinase (PI3K), MAPK and NF-*κ*B pathways, resulting in synergistic activity of the AMP and *il-1β* to recruit monocytes and macrophages to the site of infection (18).

The expression of WBC exposed to *S. molnari* shows some similarities with WBC in the bacterial assay; cytokine production is less pronounced compared to RBC, with the exception of *il6*, which is already expressed after 1 h in cells stimulated by A-2S alone. The expression of *tnfα* is not affected, while *infγ* is present in all treatments at 24 h.

The early cytokine response of WBC is *infγ* in the bacterial assay and *il6* in the parasite assay, indicating the potential for immediate antigen-processing activity (19). While IFNγ (type II interferon) exhibits antiviral and bactericidal activity against intracellular parasitic bacteria (20), IL6 promotes phagocyte proliferation in rainbow trout, but more interestingly, it upregulates interferon regulatory factor 1 (IRF1), and two trout AMPs, cathelicidin-2 and hepcidin in the head kidney macrophages (21). Due to its pleiotropic nature, IL6 is also involved in the alternative activation of macrophages and the resolution of inflammation (22), but its early upregulation in WBC in this study argues against such an anti-inflammatory role. In this case, the expression of two interacting cytokines, *il6* and *infγ*, in WBC induced by different antigens (bacterium vs parasite) suggests that A-2S adapts to an alternative pattern of TLR and cytokine activation depending on the type of co-antigen, but with a similar outcome.

When the host is infected *in vivo* with the parasite and subsequently stimulated with A-2S, the activated cytokine repertoire becomes more diverse, but consistent with the kinetics of innate immunity observed in the parasite model infection. In the latter, *S. molnari* persists in carp blood for 2-3 weeks, coinciding with the proliferation of monocytes and neutrophiles, and high *il6* and *tnfα* levels. Parasite multiplication peaks 4 weeks after infection, accompanied by lymphocytosis, but thereafter the proinflammatory conditions turn into anti-inflammatory with an exceptionally high upregulation of *il10*. This transitions the acute parasitemia into a chronic course, in which the parasite migrates from the blood to the liver (23). IL-10 primarily acts as an anti-inflammatory agent, helping to prevent overactive immune responses that can cause tissue damage, by inhibiting macrophages and dendritic cells to secrete pro-inflammatory cytokines and limiting T cell activation and proliferation. It is also a switch in the direction of proliferation, differentiation and antibody secretion by the IgM^+^ B cells, which generally attenuates an excessive immune response (24). To reverse this evolutionary suppression of inflammation, we also included a group of immunosuppressed fish (both *S. molnari*-infected and -uninfected) whose leukocyte population was depleted. As a rule of thumb, RBC showed no difference when stimulated simultaneously with the parasite and A-2S, unless the leukocyte population was depleted by cortisol. This confirms that RBC become players in the innate response and elicit an efficient proinflammatory response that is susceptible to the upregulation of A-2S. It also suggests that TLRs other than TLR4 may be affected. Indeed, AMPs can not only abrogate LPS activation of TLR4 with anti-inflammatory consequences (25) but can also activate innate signaling pathways that activate adaptive immunity by binding to bacterial dsDNA and dsRNA and consequently activating TLR9 and TLR3. The resulting nanocrystalline AMP-DNA and AMP-RNA immune complexes enter endosomal compartments and are recognized by TLR9 and TLR3, respectively (26, 27). Although anisaxins do not readily bind bacterial DNA, we have demonstrated an interaction of the peptides with plasmid DNA when applied at higher concentrations (>8x MIC) (8), similar to the concentration used here (10 µM). Considering that A-2S causes bacteriolysis within 15-20 min, it is conceivable that the fragmented bacterial dsDNA encounters A-2S, so that it forms complexes with it and eventually activates the TLR9. The most important downstream cytokines, *infγ*, *il6* and *tnfα* were recognized in this study as characteristic mediators of the proinflammatory state. Such a mechanism has been proposed for the cathelicidin LL-37 which binds to dsDNA and dsRNA and facilitates their uptake into the cytoplasm of human keratinocytes through scavenger receptors, mediating TLR9 signaling and the production of type I interferons and tnf*α* (28). While in mammals type I interferons are triggered by TLR9 activation (29), the main cytokine stimulated by TLR9 signaling in fish actually belongs to type II interferon *γ* (29), which in this study is significantly upregulated in WBC *in vivo* challenge assay.

In addition, other immune players within the *in vivo* system that contribute to the final cytokine picture observed during the 4-week parasite challenge must be considered, especially the adaptive elements regulated under TLR9 activation. Parasites such as *S. molnari* co-evolved with their hosts and have developed skillful mechanisms to evade the host immune system, maintaining the balance between host damage and the size of parasite population (30). This is clearly reflected in the cytokine repertoire of WBC which show no changes in *il6* and *il-1β*, but a consistent upregulation of *tnfα* and a clearly high *infγ* production, which increases over a period of 4 weeks. During the natural course of a *S. molnari* model infection, the first suppression of the proinflammatory response occurs after approximately 28 days, which corresponds to a peak in *il10* expression (31). Unexpectedly, in the *S. molnari in vitro* assay, changes in *il10* expression were only observed between A-2S stimulated and non-stimulated platelets (at 1 and 24 h), and not in WBC and RBC. This could be due to a higher variability in transcript quantity among WBC and RBC samples or simply due to an early timing of sampling and consequent missing of a typically delayed production of *il10* in *S. molnari* infection (31). In contrast, a general increase in *il10* was observed in fish infected with *S. molnari* at week 4 compared to earlier time points, but only in the WBC of *S. molnari* infected and A-2S-stimulated fish, consistent with a shift in *il10* response toward adaptive immunity to *S. molnari* (31). Interestingly, *il10* has also been linked to the expression of trout cathelicidins, and ability of these peptides to balance both pro- and anti-inflammatory responses depending on different cellular interactions (20).

In general, the immunomodulatory mechanisms of HDPs are diverse and play a crucial role in host defense, bridging innate and adaptive immunity, and maintaining immune homeostasis. They can either enhance or suppress immune responses depending on the context, relaying on mechanisms such as intracellular uptake, sometimes mediated by membrane-associated G protein-coupled receptors (GPCRs); interacting with several intracellular interacting protein partners or receptors (for example, GAPDH and p62); altering several signaling pathways such as nuclear factor-κB (NF-κB), p38 and JNK mitogen-activated protein kinase (MAPK), MKP1, and phospho-inositide 3-kinase (PI3K), and engaging with different transcription factors (32). While A-2S stimulates a transient proinflammatory response proceeded by an anti-inflammatory reaction, and likely engages with multiple immune cells and pathways, further research is needed to pinpoint the exact mechanism of such outcome. This is complex also because these processes appear to be dependent on factors such as the peptide concentration, the kinetics of response, and the environmental stimuli, and an *in vivo* experimental design should account for these conditions.

### 3.2 ROS production

Oxidative stress can impair the oxygen supply and induce the ageing of RBC, leading to their lysis and accumulation of erythroblast clones (31). This is consistent with our observation toward the end of the experiment: the changes in the lineage and size of the RBC were accompanied by an increase in ROS production in the RBC and WBC upon A-2S stimulation. ROS production is a recognized host defense mechanism, therefore an increase in ROS production during the *in vivo* challenge assay with *S. molnari* was expected. However, the highest ROS production was found in infected and immunosuppressed fish with a depleted lymphocyte lineage upon A-2S stimulation, additionally confirming that RBC take over the role in the innate immune response in the absence of lymphocytes.

A significant increase in ROS production, mediated by increased phagocytes uptake and phagocytosis, as well as their increased differentiation, has previously been demonstrated in dendritic cells and macrophages following stimulation by synthetic AMPs, hLF1, a human lactoferrin, and IDR-1018, respectively (33, 34).

### 3.3 Damage of the pathogens

The structural changes observed in *A. hydrophila* and *S. molnari* correspond to an unspecific A-2S effect on the cell wall and the cell membrane, respectively, which leads to plasmolysis. Roncevic et al. (8) used a 4x MIC to detect a concentration-dependent interaction with the anionic bacterial membranes. The authors found that bacterial cells were permeabilized but undisrupted, and their morphology was relatively well preserved, while molecular modeling revealed a “bulge-like” deformations of the membrane that was accompanied by lipid extraction, which eventually led to cell leakage and death.

### 3.4 Antiproliferative activity

In addition to their antimicrobial activity, some HDPs also demonstrate strong anticancer properties, commonly referred to as anti-cancer peptides (ACPs) (35). There are fundamental differences in membrane composition between normal and cancer cells, with the latter having an increased proportion of phosphatidylserine, making them negatively charged (35, 36). For this reason, and similar to the bacterial membrane, it is hypothesized that electrostatic interactions between the anionic cell membrane and the cationic peptides are responsible for the selective activity of ACPs. Certain classes of peptides have been shown to have potent anti-cancer activity, including defensins, amphibian-derived peptides such as magainin, the human cathelicidin LL-37 and some insect-derived peptides such as melittin and cecropin A and B (35). Anisaxin-2S is a cecropin-like peptide, but in contrast to the previously mentioned cecropin A and B, it has weak anti-cancer properties. It is still unclear why some peptides are active against cancer cells while others, although very similar, are not. On the other hand, the mode of action of APCs is reasonably well elucidated, so they can be categorized as membranolytic or non-membranolytic peptides. Membranolytic peptides, such as magainin, induce pore formation, leading to cell death, while non-membranolytic peptides have anti-angiogenic and anti-metastatic effects or can induce apoptosis (35). Even though anisaxin is membranolytic, it appears that induced deformations of the cell membrane and consequent leakages, do not cause inhibition of proliferation in tested tumors.

### 3.5 Antiviral activity

AMPs, including α-defensins, maximins, caerin, and indolicidin, provide protection against viruses in different ways: they bind to viral receptors expressed on the surface of the host cell membrane, preventing further viral adhesion and invasion; they act as “lectin-like” substances that target the viral glycoprotein structure; they interfere with viral RNA and protein synthesis; they disrupt the viral envelope; or they indirectly induce the expression of interferon or chemokines in the host (37, 38). In contrast, A-2S showed very limited antiviral activity against aquatic rhabdovirus (SVCV), alloherpesvirus (KHV) and paramyxovirus (CCPV). Although A-2S did not exert significant virucidal and virus entry blocking abilities in the case of the fish viruses, it did not facilitate an increase in the virus entry rate. In contrast, some AMPs, such as defensins, are known to increase infectivity of selected viruses (39). This is facilitated by enhancing viral adhesion to the cell membrane and hence internalization (40) or by increasing paracellular transport mediated through disruption of the cell-cell junction in epithelial barriers (41).

A-2S also has no direct virucidal effect on the terrestrial tick-borne virus, TBEV. We hypothesize that the virus is resilient to A-2S either due to its co-evolutionary resistance to a repertoire of AMPs produced and secreted by its tick vector (42, 43), or due to the extensive divergence of *Anisakis* spp. and the loss of function of anisaxin against terrestrial viruses.

However, A-2S is able to activate the chronic myeloid leukemia K562 cell line, as shown by the induction of *infα*, *tnfα* and *fcγr* (Fc *γ* receptor), although this activation has no effect on the replication dynamics of terrestrial TBEV. Nevertheless, we cannot exclude a possibility of an indirect effect of such activation that could lead to a reduction of the virus when other cells and mediators are involved in a more complex model in which a mutual interaction could take place, such as in a co-culture or an *in vivo* model.

Finally, limitations of the study are that the *in vitro* immunomodulation was tested at a single A-2S concentration adopted from the antimicrobial testing against *A. hydrophila*, and over a short period (0, 1, 24 h) to capture the early immune response. For more comprehensive characterization of the A-2S immunomodulatory properties, a range of concentrations could be tested, from the minimum concentration that triggers, to the maximum concentration that saturates the immune response. Additionally, the testing over longer time and in specific leukocyte lineages (i.e., antigen-presenting cells) could describe better the kinetic of the immune response, from the early pro-inflammatory and regulative anti-inflammatory response, toward its homeostasis. Based on results of A-2S pharmacokinetics in mice showing AMP’s instability, *in vivo* testing of immunomodulatory efficacy should be postponed till effective chemical modification or carrier encapsulation is developed.

## 4 Conclusions

Anisaxin-2S is a versatile nematode cecropin that has both a direct antibacterial and antiparasitic effect through its membranolytic interaction with the cell surface and an indirect effect through immunomodulation of blood lineages. The primary immunomodulation has a pro-inflammatory character, transforming into an anti-inflammatory response due to different mechanisms, contingent upon specific biological context (cell type, co-infection, time-point, etc.). This study provides the first evidence of immunomodulation of red blood cells and platelets by an AMP and describes the induction of a cytokines repertoire. However, the limitations of the study, such as longer *in vitro* assays (e.g., 3-4 days), testing of different WBC lineages, especially neutrophiles and macrophages, to narrow down the antigen-processing interaction, and assessment of the expected adaptive response, should be explored in the future. Follow up studies should continue in different directions. Firstly, a more mechanistic understanding of A-2S mode of action on the bacterial cell wall, as well as signaling pathway(s) involved in the immune response would facilitate more selective application of A-2S. In parallel, encapsulation or chemical modification of A-2S and development of a dose-response *in vivo* model would enable preclinical testing of the formulation, in particular in comparative trials with conventional antibiotics.

## 5 Materials and methods

### 5.1 Peptide synthesis

The mature region of anisaxin-2S (A-2S) from the zoonotic marine nematode *A. simplex* was synthesized by ProteoGenix (Schiltigheim, France), purified to >98% purity by RP-HPLC (LC3000, Beijing Chuangxin Tongheng Science and Technology, Beijing, China; 5 μm column, 4.6 × 250 mm) using a 25-75% acetonitrile/0.1% TFA gradient in 25 min at a flow rate of 1 mL/min (**Supplementary Figure S1**). The sequence was confirmed by ESI-MS operated in positive mode and with a flow rate of 0.2 mL/min (LCMS-2020, Shimadzu, Kyoto, Japan) (**Supplementary Figure S9**). Peptide stock solutions (3440.05 g/mol) were prepared by dissolving accurately weighed aliquots of the peptide in MiliQ water. The concentration was also verified by using the extinction coefficients at 214 nm, calculated as described by Kuipers and Gruppen (44).

### 5.2 Experimental design

The immunostimulatory properties of A-2S were first tested *in vitro* after the blood cell lineages of the common carp *Cyprinus carpio* were exposed to the Gram-negative fish bacterium *Aeromonas hydrophila* or the parasite, histozoic myxosporidian *Sphaerospora molnari* (Sphaerosporidae, Myxosporea). In a separate experiment, carp were challenged *in vivo* by *S. molnari*, and/or immunosuppressed, and their blood samples were collected over four time points and exposed to A-2S. The antiproliferative potential of A-2S was tested *in vitro* on the human bladder cancer cell line T24 and the human lung cancer cell line A549. Finally, the antiviral role of A-2S was tested *in vitro* in common carp brain cell culture (CCB) infected with a panel of fish viruses (spring viraemia of carp virus, koi herpesvirus, common carp paramyxovirus) and in the human immortalized myelogenous leukemia cell line K562 infected with tick-borne encephalitis virus (TBEV), European subtype prototypic strain Neudoerfl. The fish viruses were selected because they represent pathogens of poikilothermic organisms that occur naturally in the nematode’s environment. Therefore, if A-2S had antiviral activity, it would be noticeable. In contrast, TBEV is transmitted to homeothermic animals by a hemophagous arthropod vector, a tick, which is known to produce a variety of AMPs (45), to which TBEV may have developed an evolutionary resistance, possibly expanded also to other AMPs.

### 5.3 Experimental organism

Common carp, *Cyprinus carpio*, was reared as specific pathogen-free (SPF) from peroxide-treated fertilized eggs (700 mg/L for 15 min) in an experimental recirculation system in the animal facility of the Institute of Parasitology, Biology Centre Czech Academy of Sciences. The system has a capacity of 2,300 L of water, which flows through 40-L aquaria, equipped with a biological, mechanical and UV filtration system and ozone treatment. Every week, 25% of the water is replaced. The water quality parameters (oxygen, pH, ammonia, nitrite, and nitrates) are monitored daily using probes and titration tests. Ammonia is kept below 0.02 mg/L. The fish are kept on a constant 14 h light to 10 h dark cycle and a constant temperature of 21 ± 1°C. During the experiment, the fish were fed on a commercial carp diet (Skretting) at a daily rate of 2% of the biomass.

A total of 20 fish (weight 70 g ± 5 g, length 12 cm ± 5 cm) were randomly divided into four groups of five fish each: i) SPF fish, which served as non-treatment controls (SPF); ii) fish infected with *Sphaerospora molnari* blood stages (BS); iii) fish infected with *S. molnari* blood stages and immunosuppressed (IS+BS); and iv) uninfected, immunosuppressed fish (IS), which served as a treatment controls. Immunosuppression (IS+BS and IS groups) was achieved by intraperitoneal injection of corticosteroid (Triamcinolone acetonide, Glentham Lifesciences, 4 µL/g) to deplete lymphocyte population in the blood of the fish and exacerbate the clinical symptoms of *S. molnari* infection. This approach was adopted due to asymptomatic course of *S. molnari* infection, as the myxozoan can evade the immune system, attributed to the coevolutionary history of the parasite and fish. Therefore, clinical signs of sphaerosporosis only appear either in the case of co-infection with another pathogen or in the case of immunosuppression under controlled experimental conditions. The immunosuppression has no effect on the red blood cells in fish.

### 5.4 Blood cell culture and collection

The following protocol was used for all downstream experiments with common carp blood cells. Approximately 50 µL of blood from three to five fish per group was collected by caudal venipuncture with an insulin syringe that was rinsed with heparin before to use. The whole blood was diluted 1:2 in incomplete RPMI-1640 medium supplemented with 2% FBS (Life Technologies). Samples were placed on Ficoll separation medium and centrifuged at 500 g for 20 min at 4 °C to separate the red blood cell (RBC) and white blood cell (WBC) fractions. After the first round of Ficoll centrifugation, the RBC pellet was reloaded for yet another round of Ficoll centrifugation under the same conditions. The purity of the Ficoll-separated cell fractions was assessed by flow cytometry and light microscopy. The Ficoll-separated fractions of blood cells were counted in the Bürker chamber and adjusted to 1 x 10^6^ WBC and RBC in 1 mL of RPMI-1640 supplemented with 2% FBS. Each biological cell sample was seeded in three technical replicates in 24-well plates (Corning) and incubated at 26 °C, in 5% CO_2_ atmosphere. A-2S was used at a concentration of 10 µM (approximately equivalent to 10x MIC for *A. hydrophila*). Before the experiment, an initial sample was always taken, at 0 h (T0). After incubation (1 and 24 h), the cells were harvested from the culture plate and washed twice in RPMI-1640 by centrifugation at 500 g (5 min, 4 °C). After discarding the supernatant, RNAlater was added to the pellet and stored at 4 °C prior to RNA isolation. An additional aliquot of cells after washing in RPMI-1640 was harvested during the *S. molnari in vivo* challenge experiment (described below); these cells were placed on ice and used immediately for measurement by flow cytometry.

### 5.5 Magnetic cell sorting (MACS)

For the isolation of platelets (TC), blood samples were first incubated with a primary mouse IgM monoclonal antibody (WCL6) against common carp platelets at a ratio of 1:1, for 30 min at 4 °C (46). After washing twice in 1% FBS in PBS, the cell suspensions were incubated with secondary anti-mouse IgG MicroBeads at a ratio of 4:1 for 15 min at 4 °C, according to the MACS protocol. The platelet-enriched positive fraction was isolated by MACS LS columns (Miltenyi Biotec B.V. & Co. KG, Germany) according to the manufacturers protocol.

### 5.6 Bacterial culture

The zoonotic *Aeromonas hydrophila* was isolated and cultivated as described by Majstorović et al. (47). The isolate was cultured in the laboratory in LB agar and LB liquid medium at 37 °C for 24 h. The CFU/mL (colony forming unit) concentration was determined in the standard plate counting and correlated with the optical density OD_600_ (0.01 corresponds to 10^6^).

### 5.7 *Aeromonas hydrophila in vitro* stimulation assay

A concentration of 1 x 10^5^ and 1 x 10^6^ A*. hydrophila* in LB liquid medium was added to WBC and RBC suspensions as described above, except for the negative control. Cells were exposed to four experimental conditions: i) RPMI-1640 only (negative control); ii) A-2S (10 µM); iii) one of two *A. hydrophila* concentrations; and iv) one of two *A. hydrophila* concentrations and A-2S (10 µM) and then collected in RNAlater for RNA extraction.

### 5.8 *Sphaerospora molnari in vitro* stimulation assay

The myxozoan *Sphaerospora molnari* was isolated using the cellulose method previously described by Born-Torrilos et al. (48). Freshly isolated WBC, RBC cells and TC from five biological replicates of SPF carp were plated and co-incubation with either live *S. molnari* or anisaxin A-2S and *S. molnari* with A-2S. Cell suspensions in RPMI-1640 served as a negative control (collected at 0, 1 and 24 h). Cells were harvested in RNAlater for RNA extraction.

### 5.9 *Sphaerospora molnari in vivo* challenge followed by *in vitro* stimulation assay

RBC and WBC of fish from four experimental groups (SPF, BS, IS+BS, IS) were collected at the following time points: at the beginning of the experiment (T0), in week two (W2), in week three (W3), and in week four (W4). The latter corresponds to the peak of the *S. molnari* blood stages (parasitemia), after which the fish were humanely euthanized using an overdose (0.5 g/L) of buffered tricaine methanesulfonate (MS222; Sigma Aldrich, United States) solution in freshwater. Cells were processed as described above and harvested for RNA extraction and flow cytometry.

### 5.10 Flow cytometry

RBC and WBC were collected at each sampling point of the *in vivo* experiment with *S. molnari* to measure the production of the reactive oxygen species (ROS). In addition, an aliquot of RBC was analyzed to determine the purity of the sample and to monitor the cellular and morphological changes induced by the parasite infection. In brief, 2 µL of whole blood was washed with cold RPMI-1640 and re-suspended in 200 µL of RPMI-1640 and separated by Ficoll as described above in the “Blood cell culture and collection” section. Each sample was acquired for 20 seconds on the BD FACSCanto II (BD Biosciences) at a flow rate of 60 µL/min. RBC and WBC were identified by forward scatter-width (FSC-W)/side scatter-area (SSC-A) profile. To determine the number of apoptotic and necrotic cells, the cells were labeled with propidium iodide (PI) (ThermoFisher Scientific). ROS were measured with R123 (Dihydrorhodamine 123, λ_em_ 525 nm; ThermoFisher Scientific). The final data plots were analyzed and visualized using FlowJo™ v10.

### 5.11 Transmission electron microscopy

Additional samples of *A. hydrophila* and *S. molnari* were collected for high-pressure freezing and freeze substitution (HPF-FS) and subsequent transmission electron microscopy (TEM) to visualize the ultrastructural changes that A-2S exerts on the cell wall and cell membrane of the bacteria and the cnidarian, respectively. *Aeromonas hydrophila* 1 x 10^6^ in LB liquid medium and blood stages of *S. molnari* isolated as described above were incubated for 20 min at room temperature with A-2S 10 µM (approximately 10x MIC). The cells were then washed in PBS (centrifuged at 500 g, 5 min, 4 °C) and the pellets were processed for high-pressure freezing and freeze substitution as previously reported (49). Samples were first washed 3x in acetone for 15 min, infiltrated in 25, 50 and 75% low viscosity Spurr resin (SPI Chem, West Chester, PA, USA) in anhydrous acetone for 1 h each and incubated overnight in 100% resin. After the polymerization at 60 °C for 48 h in embedding moulds, semi-thin sections (0.5 μm) were cut from the blocks, stained with toluidine blue (1%) and checked for orientation under a light microscope. Ultrathin sections (0.07 μm) were cut from the areas of interest, mounted on Formvar-coated single-slot grids, and contrasted in ethanolic uranyl acetate (30 min) and then in lead citrate (20 min). The JEOL JEM-1400 microscope (JEOL, Akishima, Tokyo, Japan) operating at an accelerating voltage of 120 kV, was used to analyze the ultrathin sections. The XAROSA 20-megapixel CMOS camera (EMSIS GmbH) was used for imaging. The images were assembled and annotated in Inkscape 1.0 software (https://inkscape.org).

### 5.12 RNA isolation and qPCR

RNA was extracted using the NucleoSpin^®^ RNA Isolation Kit (Macherey-Nagel, Germany) according to manufacturer’s protocol. RNA concentration and purity were determined using the Nanodrop 31 ND-1000 spectrophotometer (Thermo Fisher Scientific, Wilmington, DE, USA). cDNA was synthesized from 80 ng of total RNA using the Transcriptor High Fidelity cDNA Synthesis Kit (Roche, Germany) using random hexamer primers, according to the manufacturer’s instructions. Prior to reverse transcription, the RNA was treated with DNase I (Thermo Scientific) to remove residual genomic DNA. To evaluate the innate immune response of the blood fractions stimulated by A-2S, the mRNA expression of various immunity genes was measured by qPCR (**Table 1**). Cycling conditions were set according to the QuantStudio6 SYBR Green programme (preincubation 5 min at 95 °C; 40x denaturation 30 sec at 95 °C, annealing 30 sec at 60 °C, and extension 40 sec at 72°C, with melting curve recording between 60-95 °C). cDNA was diluted 1:10 and each sample was run in duplicate. The efficiency of each primer pair, where E = 10^(− 1/slope)^, was calculated from sixfold serial dilutions of equal molar amounts of purified PCR products obtained in the PCR reaction with the respective primer pair. The efficiency of all runs was always higher than 90%, and the specificity was verified by analyzing melting curves.

**Table 1:** Characteristics of the primer sets used in this study to quantify the common carp (*Cyprinus carpio*) and human housekeeping (*) and innate immunity genes.

Target gene	Oligo sequence 5’→ 3’	Annealing Temperature (°C)	Primer efficiency (%)	Specificity	Reference
β actin*	*FW: GCTATGTGGCTCTTGACTTCGA* *RV: CCGTCAGGCAGCTCATAGCT*	FW: 56.8 RV: 59.9	99.1	fish	(58)
*il6*	*FW: CAGATAGCGGACGGAGGGGC* *RV: GCGGGTCTCTTCGTGTCTT*	FW: 63.7 RV: 57.4	94.3	fish	(59)
*il-1β*	*FW: AAGGAGGCCAGTGGCTCTGT* *RV: CCTGAAGAAGAGGAGGCTGTCA*	FW: 61.6 RV: 58.6	97.5	fish	(59)
*tnfα*	*FW: GCTGTCTGCTTCACGCTCAA* *RV: CCTTGGAAGTGACATTTGCTTTT*	FW: 58.2 RV: 53.3	94.9	fish	(60)
*il-10*	*FW: CGCCAGCATAAAGAACTCGT* *RV: TGCCAAATACTGCTCGATGT*	FW: 55.1 RV: 53.7	92.2	fish	(59)
*infγ*	*FW: CGATCAAGGAAGATGACCCAGTC* *RV: GTTGCTTCTCTGTAGACACGCTTC*	FW: 57.3 RV: 57.8	98.4	fish	(61)
β actin*	*FW: CACCATTGGCAATGAGCGGTTC* *RV: AGGTCTTTGCGGATGTCCACGT*	FW: 61.4 RV: 60.8	99.3	human	(62)
*infα*	*FW: GGCTCTAAACTCATGTAAAGAGTGCAT* *RV: AGCATGGTCATAGTTATAGCAGGG*	FW:56.3 RV: 55.9	92.8	human	(63)
*tnfα*	*FW: TCTTCTCGAACCCCGAGTGA* *RV: CCTCTGATGGCACCACCAG*	FW:57.7 RV:58.7	97.9	human	(64)
*fcγr*	*FW: GTCAAAAGTGGCGATGAGCACC* *RV: CGTGAGTAGCAAGACACCGATG*	FW: 57.3 RV: 57.7	98.1	human	OriGene, USA

### 5.13 Antiproliferative activity of A-2S

The human bladder cancer cell line T24 and the human lung cancer cell line A549 were grown in complete Dulbecco’s Modified Eagle’s Medium (DMEM; Euroclone, Italy) supplemented with 10% fetal bovine serum (FBS; Euroclone, Italy) and 1% antibiotics (penicillin and streptomycin; Euroclone, Italy) in a humidified incubator at 37 °C and 5% CO_2_. Triplicates of an identical number of cells (1 × 10^4^) were transferred to 96-well plates overnight and treated either with the complete medium (control cells, no A-2S treatment) or solutions of 10 different concentrations of A-2S for 4, 24, 48 and 72 h the next day. A-2S working solutions were prepared by dissolving the stock solution in a complete medium at final concentrations of 1, 2.5, 5, 7.5, 10, 20, 30, 50, 75 and 100 µM. Cell proliferation was determined using the MTT (3-(4,5-dimethylthiazolid-2)-2,5-diphenyltetrazoline bromide) assay that utilizes the reduction of yellow tetrazoline MTT reduction in metabolically active cells to the purple formazan. After 4, 24, 48 or 72 h of cell incubation with the different A-2S concentrations, MTT was added to all wells and incubated at 37 °C for 2 h. The reaction was stopped by removing of MTT and adding DMSO, and the plates were incubated at 37 °C for 10 min with shaking. The absorbance was measured at 570 nm using the HiPo MPP-96 microplate photometer (Biosan, Latvia). The absorbances of treated cells obtained by the MTT assay were divided by the absorbances for control cells to obtain the percentage of metabolically active cells. All samples were run in triplicate.

### 5.14 Antiviral activity of A-2S

The effect of A-2S on the infectivity of a group of fish viruses: rhabdovirus (Spring Viremia of Carp Virus - SVCV), alloherpesvirus (Koi Herpesvirus – KHV) and paramyxovirus (Common Carp Paramyxovirus - CCPV) was quantified using a 50% tissue culture infective dose (TCID_50_) assay. The assay was performed in 96-well plates with common carp brain monolayers (CCB), cultured in L-15, 10% fetal bovine serum, 0.35% glucose, 100 IU/mL penicillin and 100 mg/mL streptomycin (Merck, Germany). The cultures were incubated for 24 h at 25 °C in a humidified atmosphere before use. First, the virucidal activity of A-2S was tested: 10 µL of SVCV (1 × 10^7^ TCID_50_/mL), KHV (1 × 10^7^ TCID_50_/mL) or CCPV (5 × 10^5^ TCID_50_/mL) was mixed with 10 µl of culture medium or A-2S at the final concentrations of 100 µM, 50 µM and 10 µM, subsequently incubated at 25 °C for 18 h, and then subjected to the TCID_50_ assay. The cytopathic effect was observed for 14 days after infection. The second experiment investigated the ability of A-2S to block virus entry: 96-well plates with CCB monolayers were pre-incubated for 8 h with culture medium containing A-2S at final concentrations of 10 µM, 50 µM and 100 µM or with control medium, then the medium was removed, and the cultures were subjected to a TCID_50_ assay with SVCV, KHV or CCPV. All assays were performed in six replicates.

The human lymphoblast cell culture K-562 (CCL-243) was used for the antiviral test with tick-borne encephalitis virus, strain Neudoerfl (5th passage mouse brain). K-562 were cultured in RPMI-1640 (Biosera) with 10% FBS (Biosera), 2 mM glutamine (Biosera) and 1% antibiotic-antimycotic solution (Biosera). The cells were maintained in a humidified incubator at 37 °C and 5% CO_2_. The MTT assay was performed before the antiviral assay with K-562 and A-2S (two experiments performed in triplicate). In brief, cells (5 × 10^4^ per well) were seeded overnight in 96-well plates and treated with A-2S for 72 h the next day. A-2S working solutions were prepared by dissolving the stock solution in complete medium to a final concentration of 0, 10, 50 and 100 µM and treated as described above. For antiviral activity assays, cells (3 × 10^4^ per well) were seeded overnight in 96-well plates and treated the next day with 0, 10, 50, and 100 µM A-2S (final concentration in culture medium). After one hour, the cells were infected with TBEV Neudoerfl 0.01 MOI (Multiplicity of Infection) and placed in a humidified incubator at 37 °C with 5% CO_2_. Samples without cells were also collected to monitor the persistence of the virus in the media alone and to distinguish it from the progeny of the virus that develop in the presence of the cells. Supernatant samples were collected 1, 2 and 3 days after infection to determine the dynamics of viral replication and stored at -80 °C. Three independent experiments were performed in duplicate. The dynamics of viral replication were determined using a plaque assay according to a modified protocol by De Madrid and Porterfield (50), as previously described (51).

In addition, the K562 cell line was stimulated with A-2S prior to infection with the virus and aliquots of cells in triplicate were harvested at 1 and 24 h for measurement of human *infα*, *tnfα* and *fcγr* by qPCR as described above.

### 5.15 Statistical analyses

Normalization of the expression of the target genes for qPCR was performed on individual basis against the expression of the selected housekeeping gene (*β-actin*). Skewness and dispersion of the data were visually inspected using quantile-quantile plots, histograms and boxplots. Data were log-2 transformed to improve skewness, stabilize the variances, and achieve symmetrical treatment of up- and down- expression fold changes (52). Statistical significance of time- and A-2S-induced differences in gene expression (Log2FC) were determined by two-way ANOVA with Dunnett’s multiple comparisons *post hoc* test in Prism 9 software (GraphPad Prism 9.0, USA). The normality and homogeneity of variances of residuals were inspected by Shapiro-Wilk and Levene’s tests, respectively, and no substantial deviations were detected. Gene expression profiles were compared by hierarchical clustering on Euclidean distances and visualized using a heatmap. The heatmap was constructed by Z-score row scaling and plotted in R v4.3.2 using gplots package (53). The purpose of this was to cluster similar samples together based on similarities/difference in the expression of immunity related genes. Pairwise distances/similarities between samples were explored using multidimensional scaling (MDS) on Euclidean distances and projected into two-dimensional space. This was performed by cmdscale() function in R and plotted by ggplot2 package (54). The graphs were colored by different experimental conditions and time was shown as different plotted shapes. The reliability of the solution was verified by calculating a stress coefficient using formula seqmds.stress() from seqhandbook package for R (55). The following values were recorded: 0.1 for **Figure 1A**, 0.16 for **Figure 2A**, 0.21 for **Figure 3A**. Generally acceptable level of < 0.2 (56) was achieved in two out of three instances, but since non-metric MDS resulted in almost identical result for **Figure 3A** with much lower stress, we consider the solution reliable as well.

The differences in the antiproliferative activity of A-2S in the human bladder cancer cell line T24 and the human lung cancer cell line A549 were analyzed by unequal variances t test (GraphPad Prism 8.0, USA). IC_50_ values were calculated from the normalized data of three independent measurements of untreated controls using the same software.

The differences in the anti-viral activity of A-2S against TBEV, SVCV, KHV and CCPV were tested using non-parametric (Kruskal-Wallis) one-way ANOVA (SigmaPlot 12.5, Systat Software, USA) or the Friedman test, which is the non-parametric alternative to one-way ANOVA with repeated measures (GraphPad Prism 10.0.3, USA) (57). Prior to statistical evaluation, the data from the antiviral activity tests of A-2S against TBEV, SVCV, KHV and CCPV were transformed using Log10. The results were then tested for normal distribution (Shapiro-Wilk) and homogeneity of variance (SigmaPlot 12.5, Systat Software, USA). As they were not normally distributed, differences in antiviral activity were tested using non-Kruskal-Wallis one-way ANOVA (SigmaPlot 12.5, Systat Software, USA) or the Friedman test (GraphPad Prism 10.0.3, USA) (56). Both tests were followed by a *post hoc* Dunn’s multiple comparison test.

## Data Availability

The datasets presented in this study can be found in online repositories. The names of the repository/repositories and accession number(s) can be found in the article/**Supplementary Material**.
