# Supplementary material for: Cecropin anisaxin-2S has in vitro immunomodulatory, but not antiproliferative and antiviral properties
Source: Front Immunol. 2025 May 5;16:1567505. doi: 10.3389/fimmu.2025.1567505 (PMC12086083; doi:10.3389/fimmu.2025.1567505)
Supplement: Supplementary file 1 [file DataSheet1.docx]

Supplementary Material

# Supplementary Figures and Tables


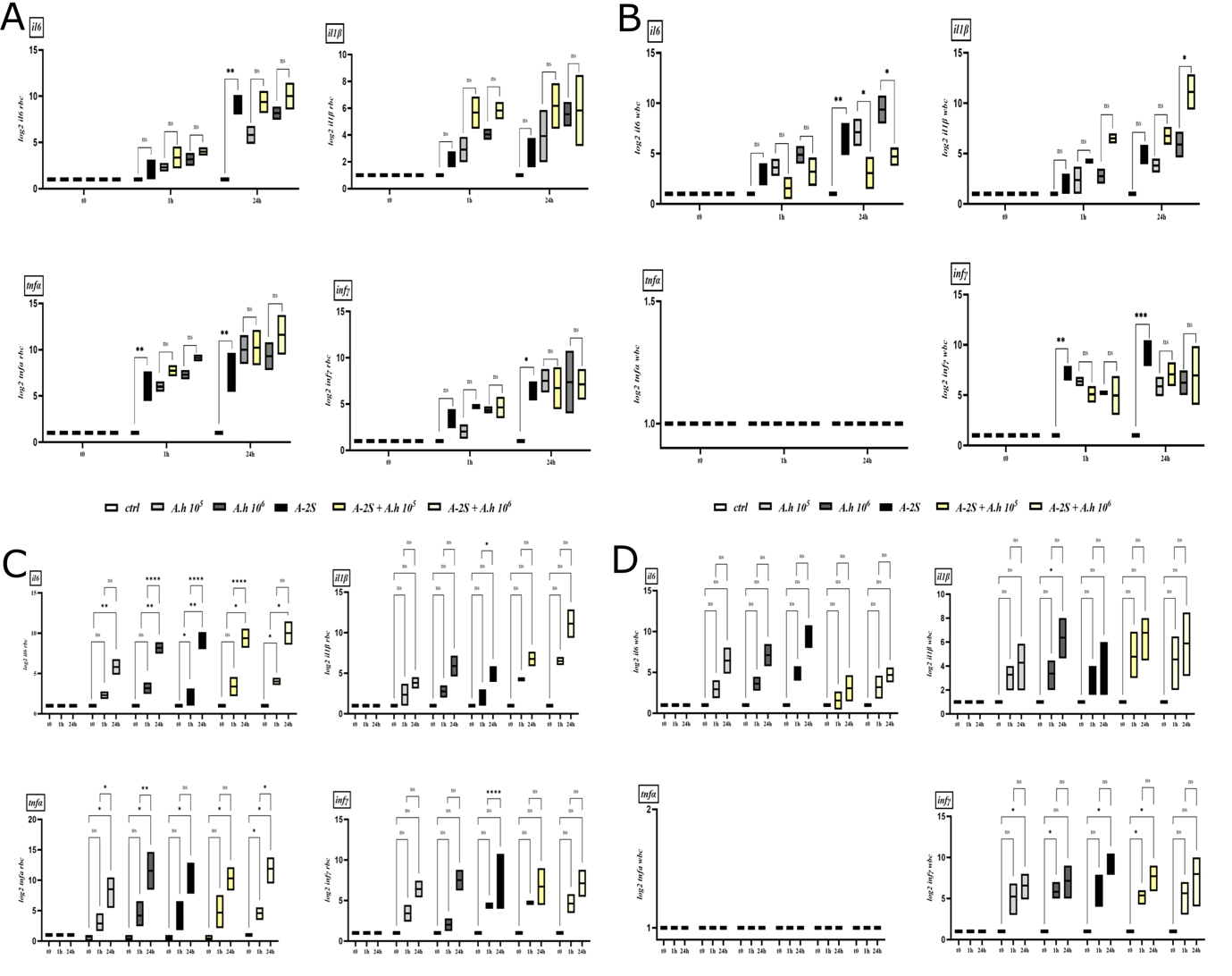


**Supplementary Figure 1 |** Graphical representation of log2-transformed fold-changes in expression of cytokines between A-2S-stimulated and unstimulated RBC (A) and WBC (B), and among time points (0, 1 and 24 h) in RBC (C) and WBC (D) priorly exposed *in vitro* to 1 x 10^5^ and 1 x 10^6^ *Aeromonas hydrophila*. CTRL: untreated cells (negative control used to calculate all fold changes); A.h 10^5^: cells exposed to 1 x 10^5^ *A. hyrdophila*; A.h 10^6^: cells exposed to 1 x 10^6^ *A. hyrdophila*; A-2S: cells stimulated by A-2S; A-2S + A.h 10^5^: cells exposed to 1 x 10^5^ *A. hyrdophila* and A-2S stimulated; A-2S + A.h 10^6^: cells exposed to 1 x 10^6^ A*. hyrdophila* and A-2S stimulated.

| **Cell type** | **treatment** |  | **cytokines** | | | |
| --- | --- | --- | --- | --- | --- | --- |
| **RBC** |  |  | ***Il6*** | ***Il-1β*** | ***tnf*𝛼** | ***inf* 𝛾** |
|  | **CTRL** |  |  |  |  |  |
|  |  | t0 vs. 1h |  |  |  |  |
|  |  | t0 vs. 24h |  |  |  |  |
|  |  | 1h vs. 24h |  |  |  |  |
|  | **A-2S** |  |  |  |  |  |
|  |  | t0 vs. 1h | 0.641 | 0.6496 | 0.181 | 0.3463 |
|  |  | t0 vs. 24h | 0.113 | 0.2096 | 0.0375* | 0.1633 |
|  |  | 1h vs. 24h | <0.0001^&^ | 0.011* | 0.0925 | <0.0001^&^ |
|  | **1 x 10^5^ *A. hydrophila*** |  |  |  |  |  |
|  |  | t0 vs. 1h | 0.2922 | 0.6496 | 0.0833 | 0.5465 |
|  |  | t0 vs. 24h | 0.1713 | 0.2104 | 0.0279* | 0.1676 |
|  |  | 1h vs. 24h | 0.1232 | 0.3592 | 0.0132* | 0.0788 |
|  | **1 x 10^5^ *A. hydrophila* + A-2S** |  |  |  |  |  |
|  |  | t0 vs. 1h | 0.3969 | 0.0667 | 0.1334 | 0.0624 |
|  |  | t0 vs. 24h | 0.1225 | 0.1329 | 0.0183* | 0.0788 |
|  |  | 1h vs. 24h | <0.0001^&^ | 0.2156 | 0.19 | 0.0788 |
|  | **1 x 10^6^ *A. hydrophila*** |  |  |  |  |  |
|  |  | t0 vs. 1h | 0.2716 | 0.3478 | 0.1172 | 0.097 |
|  |  | t0 vs. 24h | 0.0855 | 0.2211 | 0.0219* | 0.4164 |
|  |  | 1h vs. 24h | <0.001^&^ | 0.1443 | 0.0032^#^ | 0.6496 |
|  | **1 x 10^6^ *A. hydrophila* + A-2S** |  |  |  |  |  |
|  |  | t0 vs. 1h | 0.1265 | 0.0788 | 0.0421* | 0.2665 |
|  |  | t0 vs. 24h | 0.1377 | 0.1487 | 0.0206* | 0.2284 |
|  |  | 1h vs. 24h | 0.1433 | 0.3886 | 0.0223* | 0.1712 |
|  | **CRTL x CRTL + A-2S** |  |  |  |  |  |
|  |  | t0 |  |  |  |  |
|  |  | 1h | 0.9865 | 0.4198 | 0.0048^#^ | 0.8745 |
|  |  | 24h | 0.0389* | 0.7652 | 0.0037^#^ | 0.0021^#^ |
|  | **1 x 10^5^ *A. hydrophila* x 1 x 10^5^ *A. hydrophila* + A-2S** |  |  |  |  |  |
|  |  | t0 |  |  |  |  |
|  |  | 1h | 0.7845 | 0.6122 | 0.1798 | 0.2487 |
|  |  | 24h | 0.1684 | 0.4144 | 0.6442 | 0.6566 |
|  | **1 x 10^6^ *A. hydrophila* x 1 x 10^6^ *A. hydrophila* + A-2S** |  |  |  |  |  |
|  |  | t0 |  |  |  |  |
|  |  | 1h | 0.4351 | 0.1784 | 0.3447 | 0.2123 |
|  |  | 24h | 0.5697 | 0.4712 | 0.9631 | 0.4288 |
| **WBC** |  |  |  |  |  |  |
|  | **CTRL** |  |  |  |  |  |
|  |  | t0 vs. 1h |  |  |  |  |
|  |  | t0 vs. 24h |  |  |  |  |
|  |  | 1h vs. 24h |  |  |  |  |
|  | **A-2S** |  |  |  |  |  |
|  |  | t0 vs. 1h | 0.4368 | 0.1838 |  | 0.0707 |
|  |  | t0 vs. 24h | 0.2463 | 0.2377 |  | 0.0128* |
|  |  | 1h vs. 24h | 0.1232 | 0.3339 |  | 0.2816 |
|  | **1 x 10^5^ *A. hydrophila*** |  |  |  |  |  |
|  |  | t0 vs. 1h | 0.2725 | 0.1119 |  | 0.102 |
|  |  | t0 vs. 24h | 0.1889 | 0.1649 |  | 0.0391* |
|  |  | 1h vs. 24h | 0.1232 | 0.3339 |  | 0.7345 |
|  | **1 x 10^5^ *A. hydrophila* + A-2S** |  |  |  |  |  |
|  |  | t0 vs. 1h | 0.8526 | 0.12 |  | 0.0246* |
|  |  | t0 vs. 24h | 0.5543 | 0.0579 |  | 0.0293* |
|  |  | 1h vs. 24h | 0.2777 | 0.4636 |  | 0.2862 |
|  | **1 x 10^6^ *A. hydrophila*** |  |  |  |  |  |
|  |  | t0 vs. 1h | 0.1933 | 0.1255 |  | 0.0236* |
|  |  | t0 vs. 24h | 0.1417 | 0.0481* |  | 0.0522 |
|  |  | 1h vs. 24h | 0.0961 | 0.2816 |  | 0.1256 |
|  | **1 x 10^6^ *A. hydrophila* + A-2S** |  |  |  |  |  |
|  |  | t0 vs. 1h | 0.4836 | 0.1761 |  | 0.108 |
|  |  | t0 vs. 24h | 0.2089 | 0.1304 |  | 0.1091 |
|  |  | 1h vs. 24h | 0.2777 | 0.7148 |  | 0.1115 |
|  | **CRTL x CRTL + A-2S** |  |  |  |  |  |
|  |  | t0 |  |  |  |  |
|  |  | 1h | 0.4763 | 0.4412 |  | 0.0188* |
|  |  | 24h | 0.0045^#^ | 0.4587 |  | 0.0082^#^ |
|  | **1 x 10^5^ *A. hydrophila* x 1 x 10^5^ *A. hydrophila* + A-2S** |  |  |  |  |  |
|  |  | t0 |  |  |  |  |
|  |  | 1h | 0.2789 | 0.3974 |  | 0.6578 |
|  |  | 24h | 0.0026^#^ | 0.4412 |  | 0.1156 |
|  | **1 x 10^6^ *A. hydrophila* x 1 x 10^6^ *A. hydrophila* + A-2S** |  |  |  |  |  |
|  |  | t0 |  |  |  |  |
|  |  | 1h | 0.7779 | 0.1498 |  | 0.4338 |
|  |  | 24h | 0.0198* | 0.0025^#^ |  | 0.1479 |
| RBC: red blood cells; WBC: white blood cells; A-2S: anisaxin 2S; CRTL: control; *il6*: interleukin 6; *Il-1β*: Interleukin 1β; *tnf*𝛼: tumor necrosis factor 𝛼; *inf* 𝛾: interferon 𝛾. *p < 0.05; ^#^p < 0.01; ^$^p < 0.001 and ^&^p < 0.0001 | | | | | | |
|  | | | | | | |

**Supplementary Table 1 |** Statistical significance of the difference in cytokines’ log2-transformed fold-changes (calculated relative to the control SPF group) among time points (0, 1 and 24 h) and between A-2S-stimulated and unstimulated cells priorly exposed *in vitro* to 1 x 10^5^ and 1 x 10^6^ *Aeromonas hydrophila* obtained by two-way ANOVA performed with Dunnett’s multiple comparisons post hoc test.


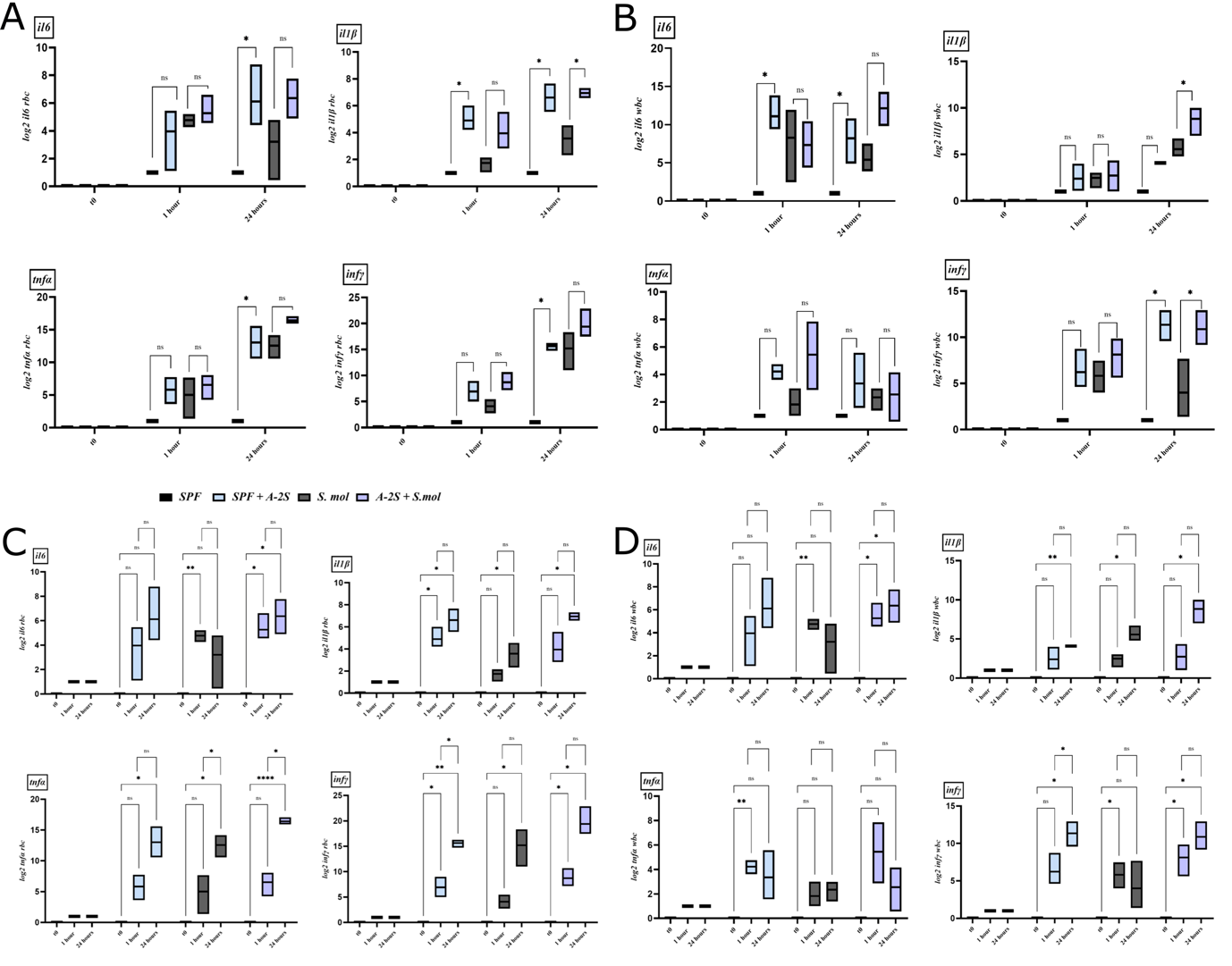


**Supplementary Figure 2 |** Graphical representation of log2-transformed fold-changes in expression of cytokines between A-2S-stimulated and unstimulated RBC (A) and WBC (B) and among time points (0, 1 and 24 h) in RBC (C) and WBC (D) priorly exposed *in vitro* to *Sphaerospora molnari*. SPF: untreated cells (negative control used to calculate all fold changes); SPF + A-2S: cells stimulated by A-2S; S. mol: cells exposed to *S. molnari*; A-2S + S. mol: cells exposed to *S. molnari* and A-2S-stimulated.

**
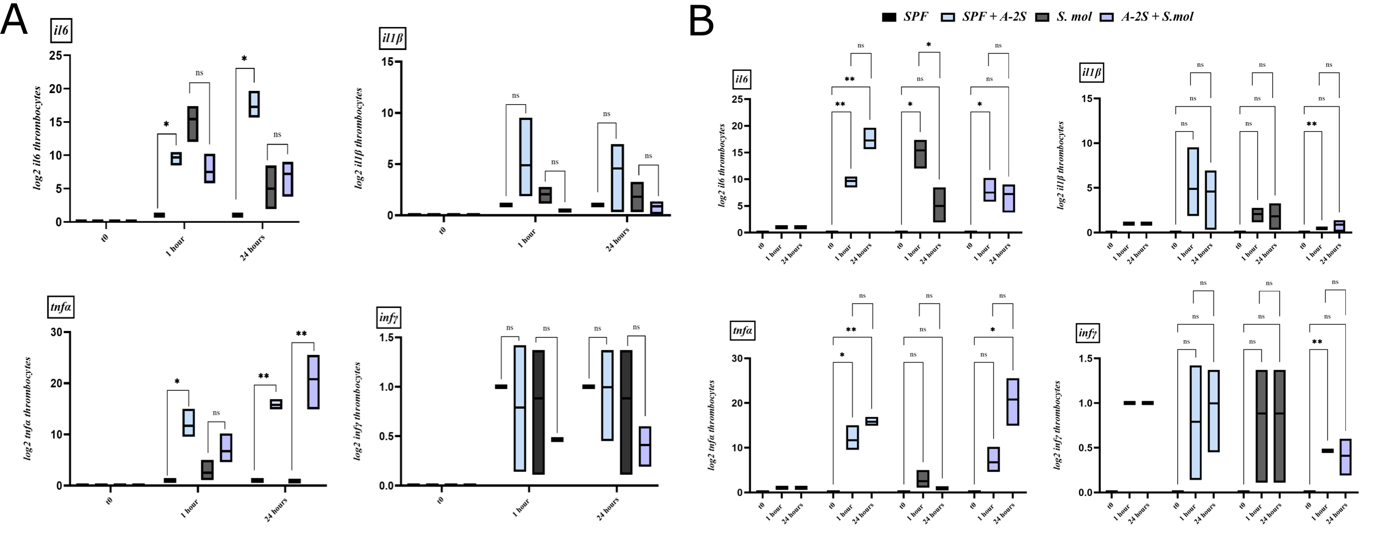
**

**Supplementary Figure 3 |** Graphical representation of log2-transformed fold-changes in expression of cytokines in platelets between A-2S-stimulated and unstimulated cells (A) and among time points (0, 1 and 24 h) (B) priorly exposed *in vitro* to *Sphaerospora molnari*. SPF: untreated cells (negative control used to calculate all fold changes); SPF + A-2S: cells stimulated by A-2S; S. mol: cells exposed to *S. molnari*; A-2S + S. mol: cells exposed to *S. molnari* and A-2S-stimulated.

| **Cell type** | **treatment** |  | **cytokines** | | | |
| --- | --- | --- | --- | --- | --- | --- |
| **RBC** |  |  | ***Il6*** | ***Il-1β*** | ***tnf*𝛼** | ***inf* 𝛾** |
|  | **SPF** |  |  |  |  |  |
|  |  | t0 vs. 1h |  |  |  |  |
|  |  | t0 vs. 24h |  |  |  |  |
|  |  | 1h vs. 24h |  |  |  |  |
|  | **SPF + A-2S** |  |  |  |  |  |
|  |  | t0 vs. 1h | 0.1657 | 0.02* | 0.072 | 0.0414* |
|  |  | t0 vs. 24h | 0.0703 | 0.013* | 0.0221* | 0.0014^#^ |
|  |  | 1h vs. 24h | 0.0313* | 0.2802 | 0.1444 | 0.0256* |
|  | ***S. molnari*** |  |  |  |  |  |
|  |  | t0 vs. 1h | 0.0052^#^ | 0.0579 | 0.204 | 0.0549 |
|  |  | t0 vs. 24h | 0.2212 | 0.0497* | 0.0127* | 0.0308* |
|  |  | 1h vs. 24h | 0.3257 | 0.0588 | 0.0425* | 0.1256 |
|  | ***S. molnari* + A-2S** |  |  |  |  |  |
|  |  | t0 vs. 1h | 0.0243* | 0.0632 | 0.0544 | 0.0216* |
|  |  | t0 vs. 24h | 0.0257* | 0.0435* | <0.0001^&^ | 0.0123* |
|  |  | 1h vs. 24h | 0.2185 | 0.2738 | 0.0208* | 0.1985 |
|  | **SPF x SPF + A-2S** |  |  |  |  |  |
|  |  | t0 |  |  |  |  |
|  |  | 1h | 0.3565 | 0.0031^#^ | 0.1715 | 0.1605 |
|  |  | 24h | 0.0023^#^ | 0.0037^#^ | 0.0029^#^ | 0.0218* |
|  | ***S. molnari* x *S. molnari* + A-2S** |  |  |  |  |  |
|  |  | t0 |  |  |  |  |
|  |  | 1h | 0.2485 | 0.4123 | 0.1896 | 0.313 |
|  |  | 24h | 0.1897 | 0.0274* | 0.2221 | 0.2541 |
| **WBC** |  |  |  |  |  |  |
|  | **SPF** |  |  |  |  |  |
|  |  | t0 vs. 1h |  |  |  |  |
|  |  | t0 vs. 24h |  |  |  |  |
|  |  | 1h vs. 24h |  |  |  |  |
|  | **SPF + A-2S** |  |  |  |  |  |
|  |  | t0 vs. 1h | 0.0237* | 0.1641 | 0.0093^#^ | 0.3061 |
|  |  | t0 vs. 24h | 0.0654 | 0.0015^#^ | 0.1584 | 0.0113* |
|  |  | 1h vs. 24h | 0.1256 | 0.1456 | 0.1785 | 0.0289* |
|  | ***S. molnari*** |  |  |  |  |  |
|  |  | t0 vs. 1h | 0.1619 | 0.0703 | 0.1434 | 0.044* |
|  |  | t0 vs. 24h | 0.0588 | 0.0169* | 0.0631 | 0.2509 |
|  |  | 1h vs. 24h | 0.2156 | 0.2113 | 0.1365 | 0.1964 |
|  | ***S. molnari* + A-2S** |  |  |  |  |  |
|  |  | t0 vs. 1h | 0.0815 | 0.1602 | 0.0973 | 0.037* |
|  |  | t0 vs. 24h | 0.0172* | 0.0167* | 0.2064 | 0.0156* |
|  |  | 1h vs. 24h | 0.1775 | 0.2313 | 0.0945 | 0.0231* |
|  | **SPF x SPF + A-2S** |  |  |  |  |  |
|  |  | t0 |  |  |  |  |
|  |  | 1h | 0.0028^#^ | 0.2578 | 0.909 | 0.6312 |
|  |  | 24h | 0.0014^#^ | 0.1878 | 0.9142 | 0.0034^#^ |
|  | ***S. molnari* x *S. molnari* + A-2S** |  |  |  |  |  |
|  |  | t0 |  |  |  |  |
|  |  | 1h | 0.1414 | 0.2415 | 0.7412 | 0.4912 |
|  |  | 24h | 0.2314 | 0.0034^#^ | 0.5542 | 0.0019^#^ |
| **TC** |  |  |  |  |  |  |
|  | **SPF** |  |  |  |  |  |
|  |  | t0 vs. 1h |  |  |  |  |
|  |  | t0 vs. 24h |  |  |  |  |
|  |  | 1h vs. 24h |  |  |  |  |
|  | **SPF + A-2S** |  |  |  |  |  |
|  |  | t0 vs. 1h | 0.0063^#^ | 0.2601 | 0.0312* | 0.2493 |
|  |  | t0 vs. 24h | 0.0077^#^ | 0.2478 | 0.0022^#^ | 0.1078 |
|  |  | 1h vs. 24h | 0.0803 | 0.9965 | 0.2867 | 0.9219 |
|  | ***S. molnari*** |  |  |  |  |  |
|  |  | t0 vs. 1h | 0.0188* | 0.0755 | 0.2708 | 0.2294 |
|  |  | t0 vs. 24h | 0.1813 | 0.2495 | 0.1078 | 0.2294 |
|  |  | 1h vs. 24h | 0.0232* | 0.8987 | 0.419 | 0.0569 |
|  | ***S. molnari* + A-2S** |  |  |  |  |  |
|  |  | t0 vs. 1h | 0.0497* | 0.0017^#^ | 0.093 | 0.0017^#^ |
|  |  | t0 vs. 24h | 0.0799 | 0.2294 | 0.0338* | 0.1151 |
|  |  | 1h vs. 24h | 0.9703 | 0.5524 | 0.0681 | 0.909 |
|  | **SPF x SPF + A-2S** |  |  |  |  |  |
|  |  | t0 |  |  |  |  |
|  |  | 1h | 0.0041^#^ | 0.3875 | 0.0056^#^ | 0.9856 |
|  |  | 24h | 0.0022^#^ | 0.7788 | 0.0024^#^ | 0.7895 |
|  | ***S. molnari* x *S. molnari* + A-2S** |  |  |  |  |  |
|  |  | t0 |  |  |  |  |
|  |  | 1h | 0.1699 | 0.9633 | 0.1018 | 0.7452 |
|  |  | 24h | 0.3255 | 0.4554 | 0.0072^#^ | 0.4361 |
| RBC: red blood cells; WBC: white blood cells; TC: platelets; A-2S: anisaxin 2S; SPF: specific pathogen free; *il6*: interleukin 6; *Il-1β*: Interleukin 1β; *tnf*𝛼: tumor necrosis factor 𝛼; *inf* 𝛾: interferon 𝛾. *p < 0.05; ^#^p < 0.01; ^$^p < 0.001 and ^&^p < 0.0001 | | | | | | |
|  | | | | | | |

**Supplementary Table 2 |** Statistical significance of the difference in cytokines’ log2-transformed fold-changes (calculated relative to the control SPF group) among time points (0, 1 and 24 h) and between A-2S-stimulated and unstimulated cells priorly exposed *in vitro* to the cnidarian *Sphaerospora molnari* obtained by two-way ANOVA performed with Dunnett’s multiple comparisons post hoc test.


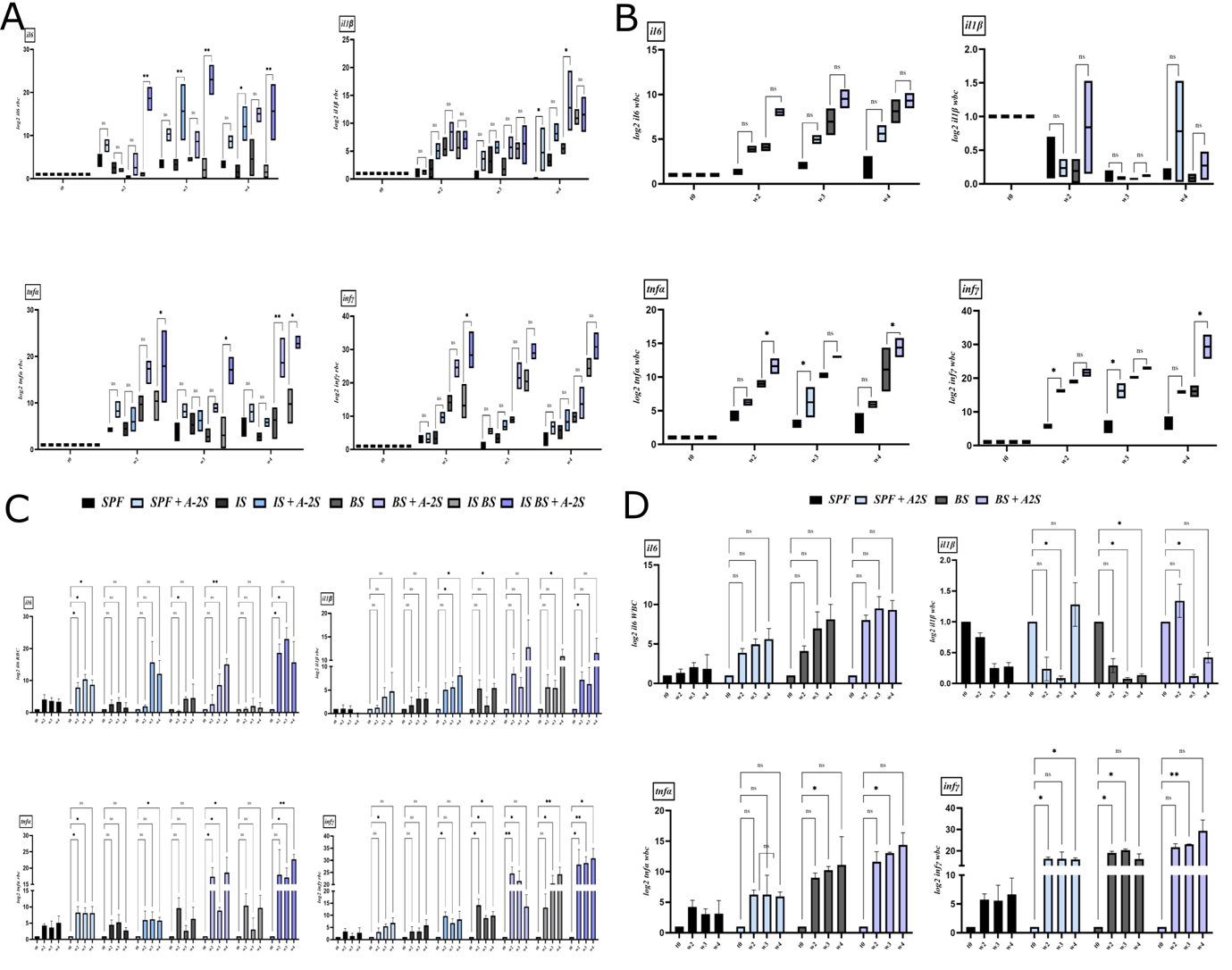


**Supplementary Figure 4 |** Graphical representation of log2-transformed fold-changes in expression of cytokines between A-2S-stimulated and unstimulated RBC (A) and WBC (B) and among time points (0, 2, 3 and 4 week) in RBC (C) and WBC (D) priorly *in vivo* challenged by *Sphaerospora molnari*. SPF: untreated cells (negative control used to calculate all fold changes); SPF + A-2S: cells stimulated by A-2S; IS: immunosuppressed cells; IS + A-2S: immunosuppressed cells stimulated by A-2S; BS: cells infected by *S. molnari* blood stages; BS + A-2S: cells infected by *S. molnari* blood stages and A-2S-stimulated; IS BS: immunosuppressed cells infected by *S. molnari* blood stages; IS BS + A-2S: immunosuppressed cells infected by *S. molnari* blood stages and A-2S-stimulated.

| **Cell type** | **treatment** |  | **cytokines** | | | |
| --- | --- | --- | --- | --- | --- | --- |
| **RBC** |  |  | ***Il6*** | ***Il-1β*** | ***tnf*𝛼** | ***inf* 𝛾** |
|  | **SPF** |  |  |  |  |  |
|  |  | t0 vs. w2 | 0.1391 | >0.999 | 0.0137* | 0.1691 |
|  |  | t0 vs. w3 | 0.0935 | 0.9954 | 0.271 | 0.8552 |
|  |  | t0 vs. w4 | 0.0833 | 0.0036^#^ | 0.1474 | 0.4946 |
|  | **SPF + A-2S** |  |  |  |  |  |
|  |  | t0 vs. w2 | 0.0313* | 0.6818 | 0.0426* | 0.3007 |
|  |  | t0 vs. w3 | 0.0169* | 0.2615 | 0.031* | 0.0285* |
|  |  | t0 vs. w4 | 0.0249* | 0.4393 | 0.051 | 0.0776 |
|  | **IS** |  |  |  |  |  |
|  |  | t0 vs. w2 | 0.3353 | 0.78 | 0.1098 | 0.354 |
|  |  | t0 vs. w3 | 0.1861 | 0.4653 | 0.1658 | 0.2199 |
|  |  | t0 vs. w4 | 0.8958 | 0.1744 | 0.1517 | 0.1356 |
|  | **IS + A-2S** |  |  |  |  |  |
|  |  | t0 vs. w2 | 0.1917 | 0.0766 | 0.1741 | 0.0253* |
|  |  | t0 vs. w3 | 0.1143 | 0.0395* | 0.1099 | 0.0544 |
|  |  | t0 vs. w4 | 0.0843 | 0.0342* | 0.0253* | 0.125 |
|  | **BS** |  |  |  |  |  |
|  |  | t0 vs. w2 | 0.1509 | 0.1085 | 0.0795 | 0.0232* |
|  |  | t0 vs. w3 | 0.019* | 0.8268 | 0.3686 | 0.0105* |
|  |  | t0 vs. w4 | 0.5051 | 0.0307 | 0.2283 | 0.0224* |
|  | **BS + A-2S** |  |  |  |  |  |
|  |  | t0 vs. w2 | 0.7257 | 0.095 | 0.0188* | 0.0085^#^ |
|  |  | t0 vs. w3 | 0.1147 | 0.1107 | 0.0105* | 0.0262* |
|  |  | t0 vs. w4 | 0.01^#^ | 0.1365 | 0.0443* | 0.10884 |
|  | **IS BS** |  |  |  |  |  |
|  |  | t0 vs. w2 | 0.9708 | 0.1799 | 0.0908 | 0.1214 |
|  |  | t0 vs. w3 | 0.8283 | 0.0813 | 0.6834 | 0.0188* |
|  |  | t0 vs. w4 | 0.8958 | 0.0137* | 0.1097 | 0.0098^#^ |
|  | **IS BS + A-2S** |  |  |  |  |  |
|  |  | t0 vs. w2 | 0.0155* | 0.0466* | 0.1197 | 0.0319* |
|  |  | t0 vs. w3 | 0.0158* | 0.2223 | 0.0211* | 0.0054^#^ |
|  |  | t0 vs. w4 | 0.1143 | 0.0544 | 0.10031 | 0.0119* |
|  | **SPF x SPF + A-2S** |  |  |  |  |  |
|  |  | w2 | 0.5684 | 0.3218 | 0.2926 | 0.3441 |
|  |  | w3 | 0.36354 | 0.4785 | 0.746 | 0.8674 |
|  |  | w4 | 0.2449 | 0.0393* | 0.112 | 0.1775 |
|  | **IS x IS + A-2S** |  |  |  |  |  |
|  |  | w2 | 0.2256 | 0.1786 | 0.9974 | 0.5541 |
|  |  | w3 | 0.1593 | 0.3985 | 0.1064 | 0.313 |
|  |  | w4 | 0.0747 | 0.4128 | 0.5304 | 0.9184 |
|  | **BS x BS + A-2S** |  |  |  |  |  |
|  |  | w2 | 0.8954 | 0.1444 | 0.8875 | 0.3365 |
|  |  | w3 | 0.0047^#^ | 0.2807 | 0.2543 | 0.4362 |
|  |  | w4 | 0.8501 | 0.0364* | 0.0489* | 0.8784 |
|  | **IS BS x IS BS + A-2S** |  |  |  |  |  |
|  |  | w2 | 0.0255* | 0.2496 | 0.0645 | 0.2159 |
|  |  | w3 | 0.0149* | 0.6981 | 0.0278* | 0.4754 |
|  |  | w4 | 0.1114 | 0.446 | 0.0038^#^ | 0.0048^#^ |
| **WBC** |  |  |  |  |  |  |
|  | **SPF** |  |  |  |  |  |
|  |  | t0 vs. w2 | 0.723 | 0.4582 | 0.236 | 0.1511 |
|  |  | t0 vs. w3 | 0.3626 | 0.0959 | 0.2844 | 0.3866 |
|  |  | t0 vs. w4 | 0.8575 | 0.0993 | 0.5825 | 0.3368 |
|  | **SPF + A-2S** |  |  |  |  |  |
|  |  | t0 vs. w2 | 0.1313 | 0.1745 | 0.0987 | 0.0342* |
|  |  | t0 vs. w3 | 0.1235 | 0.0274* | 0.3965 | 0.1462 |
|  |  | t0 vs. w4 | 0.204 | 0.977 | 0.104 | 0.0346* |
|  | **BS** |  |  |  |  |  |
|  |  | t0 vs. w2 | 0.1451 | 0.1224 | 0.0676 | 0.0301* |
|  |  | t0 vs. w3 | 0.2449 | 0.0163* | 0.0449* | 0.0216* |
|  |  | t0 vs. w4 | 0.1848 | 0.0174* | 0.3094 | 0.1144 |
|  | **BS + A-2S** |  |  |  |  |  |
|  |  | t0 vs. w2 | 0.0642 | 0.494 | 0.1096 | 0.0567 |
|  |  | t0 vs. w3 | 0.1231 | 0.0228* | 0.0106* | 0.0057^#^ |
|  |  | t0 vs. w4 | 0.1014 | 0.1033 | 0.1057 | 0.1257 |
|  | **SPF x SPF + A-2S** |  |  |  |  |  |
|  |  | w2 | 0.7575 | 0.1588 | 0.7875 | 0.0024 |
|  |  | w3 | 0.1414 | 0.4756 | 0.7635 | 0.0031 |
|  |  | w4 | 0.5485 | 0.3845 | 0.0034 | 0.7856 |
|  | **BS x BS + A-2S** |  |  |  |  |  |
|  |  | w2 | 0.7489 | 0.8896 | 0.0015^#^ | 0.4568 |
|  |  | w3 | 0.1145 | 0.7325 | 0.6654 | 0.4351 |
|  |  | w4 | 0.4445 | 0.9693 | 0.0023^#^ | 0.0042^#^ |
| RBC: red blood cells; WBC: white blood cells; BS: blood stages of *Sphaerospora molnari*; IS: cortisol-immunosuppressed fish; IS BS: cortisol-immunosuppressed fish infected by blood stages of *Sphaerospora molnari*; A-2S: anisaxin 2S; SPF: specific pathogen free; *il6*: interleukin 6; *Il-1β*: Interleukin 1β; *tnf*𝛼: tumor necrosis factor 𝛼; *inf* 𝛾: interferon 𝛾. *p < 0.05; ^#^p < 0.01; ^$^p < 0.001 and ^&^p < 0.0001 | | | | | | |

**Supplementary Table 3 |** Statistical significance of the difference in log2-transformed fold-changes of cytokines (calculated relative to the control SPF group) among time points (0, 1 and 24 h) and between A-2S-stimulated and unstimulated cells isolated from fish *in vivo* infected by cnidarian *Sphaerospora molnari* obtained by two-way ANOVA performed with Dunnett’s multiple comparisons post hoc test

**Supplementary Figure 5 |** Graphical representation of log2-transformed fold-changes in *il10* expression among time points (0, 1, 24 h) in RBC (A), WBC (B) and platelets (C); and between A-2S-stimulated and unstimulated RBC (D), WBC (E) and platelets (F) priorly exposed *in vitro* to *Sphaerospora molnari*. As well as graphical representation of log2-transformed fold-changes in *il10* expression among time points (0, 2, 3 and 4 week) in RBC (G) and WBC (I), and between A-2S-stimulated and unstimulated RBC (H) and WBC (J) priorly *in vivo* challenged by *Sphaerospora molnari*. SPF: untreated cells (negative control used to calculate all fold changes); SPF + A-2S: cells stimulated by A-2S; IS: immunosuppressed cells; IS + A-2S: immunosuppressed cells stimulated by A-2S; BS: cells infected by *S. molnari* blood stages; BS + A-2S: cells infected by *S. molnari* blood stages and A-2S-stimulated; IS BS: immunosuppressed cells infected by *S. molnari* blood stages; IS BS + A-2S: immunosuppressed cells infected by *S. molnari* blood stages and A-2S-stimulated.


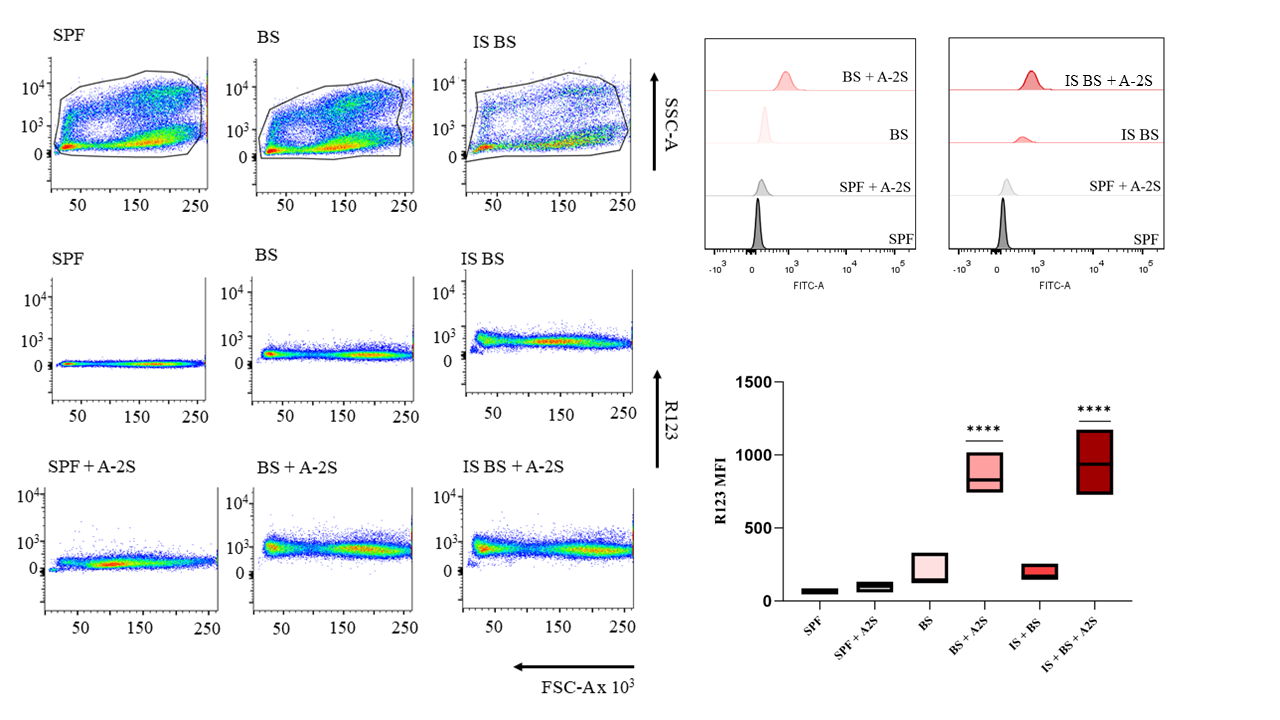


**Supplementary Figure 6 |** Dihydrorhodamine 123 (R123)-positive RBC analyzed by flow cytometry, and visualized plotted as R123 fluorescence (y-axis) versus forward scatter area (FSC-A) (*left*). RBC were collected from healthy fish (SPF), *Sphaerospora molnari*-infected fish (BS) and *S. molnari*-infected and immunosuppressed fish (IS BS) at fourth week of *in vivo* challenge, and compared to RBC collected from the same experimental groups, additionally treated with A-2S (SP+A-2S, BS+A-2S, IS BS + A-2S). *On the right*: Representative histograms of the flow cytometry data presented on left, including the summary of the data (*right, bottom*): MFI (mean fluorescence intensity) for all experimental groups at the tested time-points. Data are presented as box plots, where the middle line represents the median. A two-way ANOVA was performed with Dunnett’s multiple comparisons *post hoc* test to compare each experimental condition to its respective t0 SPF group at each time-point. **** p < 0.0001.

**
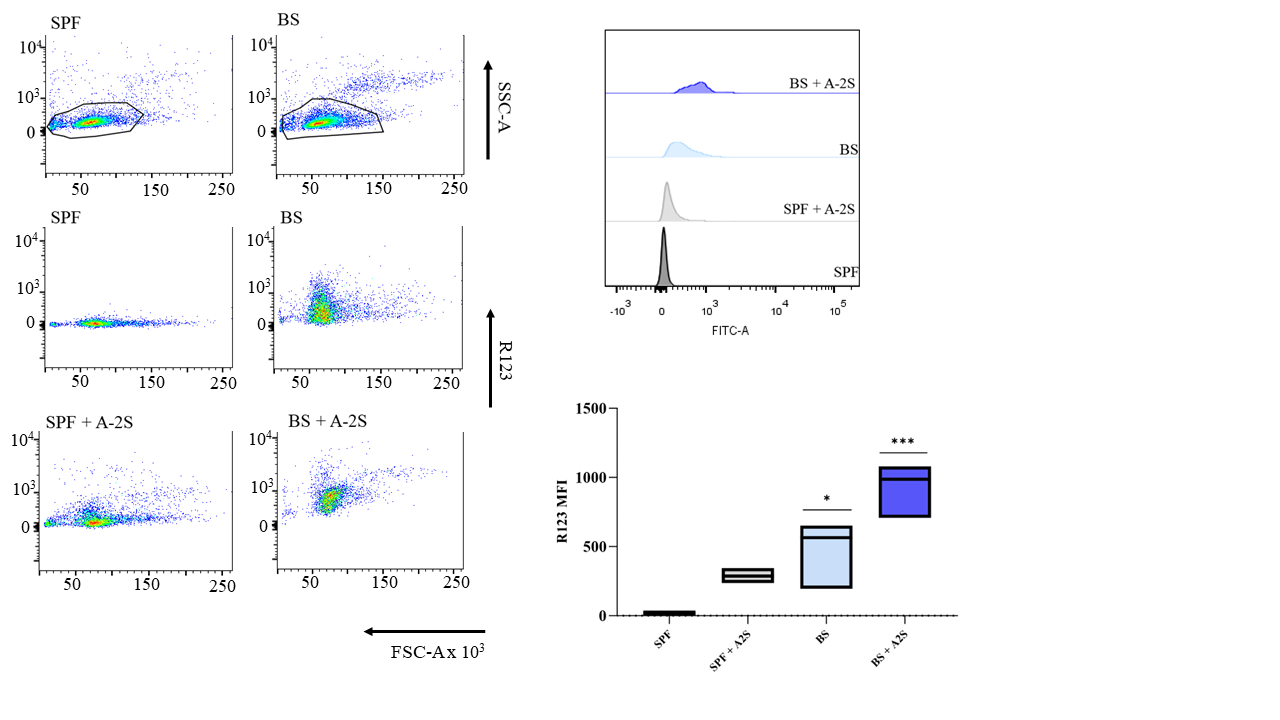
**

**Supplementary Figure 7 |** Dihydrorhodamine 123 (R123)-positive WBC analyzed by flow cytometry, and visualized plotted as R123 fluorescence (y-axis) versus forward scatter area (FSC-A) (*left*). WBC were collected from healthy fish (SPF), *Sphaerospora molnari*-infected fish (BS) and *S. molnari*-infected and immunosuppressed fish (IS BS) at fourth week of *in vivo* challenge, and compared to WBC collected from the same experimental groups, additionally treated with A-2S (SP+A-2S, BS+A-2S, IS BS + A-2S). *On the right*: Representative histograms of the flow cytometry data presented on left, including the summary of the data (*right, bottom*): MFI (mean fluorescence intensity) for all experimental groups at the tested time-points. Data are presented as box plots, where the middle line represents the median. A two-way ANOVA was performed with Dunnett’s multiple comparisons *post hoc* test to compare each experimental condition to its respective t0 SPF group at each time-point. * p < 0.05, *** p < 0.001.

**
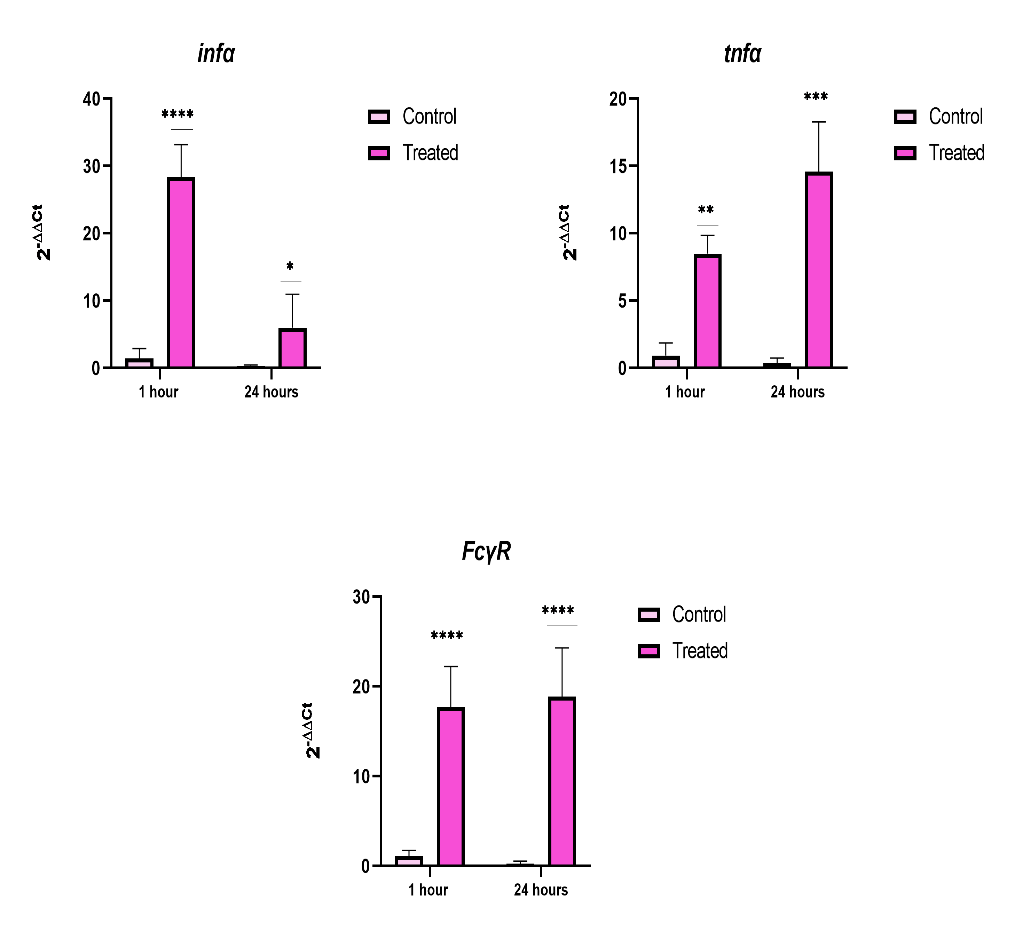
**

**Supplementary Figure 8 |** Graphical representation of differences in *inf𝛼*, *tnf𝛼* and *fc𝛾r* expression between A-2S stimulated (Treated) and unstimulated (Control) K562 chronic myelogenous leukemia cells at 1 and 24 h post-treatment.

# Supplementary Figure 9 | Anisaxin-2S mass spectrometry report.
